# Early-Life Events, Including Mode of Delivery and Type of Feeding, Siblings and Gender, Shape the Developing Gut Microbiota

**DOI:** 10.1371/journal.pone.0158498

**Published:** 2016-06-30

**Authors:** Rocio Martin, Hiroshi Makino, Aysun Cetinyurek Yavuz, Kaouther Ben-Amor, Mieke Roelofs, Eiji Ishikawa, Hiroyuki Kubota, Sophie Swinkels, Takafumi Sakai, Kenji Oishi, Akira Kushiro, Jan Knol

**Affiliations:** 1 Nutricia Research, Utrecht, The Netherlands; 2 Yakult Central Institute, Tokyo, Japan; 3 Laboratory of Microbiology Wageningen University, Wageningen, The Netherlands; GI Lab, UNITED STATES

## Abstract

Colonization of the infant gut is believed to be critically important for a healthy growth as it influences gut maturation, metabolic, immune and brain development in early life. Understanding factors that influence this process is important, since an altered colonization has been associated with a higher risk of diseases later in life. Fecal samples were collected from 108 healthy neonates in the first half year of life. The composition and functionality of the microbiota was characterized by measuring 33 different bacterial taxa by qPCR/RT qPCR, and 8 bacterial metabolites. Information regarding gender, place and mode of birth, presence of siblings or pets; feeding pattern and antibiotic use was collected by using questionnaires. Regression analysis techniques were used to study associations between microbiota parameters and confounding factors over time. Bacterial DNA was detected in most meconium samples, suggesting bacterial exposure occurs *in utero*. After birth, colonization by species of *Bifidobacterium*, *Lactobacillus* and *Bacteroides* was influenced by mode of delivery, type of feeding and presence of siblings, with differences found at species level and over time. Interestingly, infant-type bifidobacterial species such as *B*. *breve* or *B*. *longum* subsp *infantis* were confirmed as early colonizers apparently independent of the factors studied here, while *B*. *animalis subsp*. *lactis* presence was found to be dependent solely on the type of feeding, indicating that it might not be a common infant gut inhabitant. One interesting and rather unexpected confounding factor was gender. This study contributes to our understanding of the composition of the microbiota in early life and the succession process and the evolution of the microbial community as a function of time and events occurring during the first 6 months of life. Our results provide new insights that could be taken into consideration when selecting nutritional supplementation strategies to support the developing infant gut microbiome.

## Introduction

Composed of 10^11^ to 10^12^ micro-organisms per gram of feces, and estimated to harbor more than 500 bacterial species, the human gastrointestinal microbiota is a large and diverse community of microorganisms and is in close cross-talk with its host [1–3]. Despite the vast number of bacteria and complexity found in the adult gut, the microbiota of the infant gut is initially a simple ecosystem which gradually undergoes successional changes until it reaches high diversity. For years, the infant gut was considered to be sterile, only becoming colonized after birth from the maternal microbiota, diet, and the environment [4–7]. However, recent findings suggest that microbial exposure may start already during gestation and colonization starts with the early settlers derived from the maternal microbiota and environment immediately from birth [8–11]. The development of the infant gut microbiota is profoundly influenced by host genotype, gestational age, antibiotic use, mode of delivery, diet and the context in which the infant is born (rural vs urban, presence of siblings, pets and other factors) [4, 5, 12–17].

The first colonizers of the infant gut are facultative anaerobic bacteria, such as *Staphylococcus*, *Streptococcus*, *Enterococcus* and *Enterobacter* spp, which by reducing the oxygen levels that initially exist, pave the way for anaerobes such as *Bifidobacterium*, *Bacteroides* and *Clostridium* spp. to start colonizing the gut. It is known that the type of bacteria that initially colonize the infant gut and the time frame in which this occurs, is highly dependent on the mode of delivery. Previous studies have demonstrated that strains originating from the maternal gut and vagina are transferred to the infant’s gut in case of a vaginal delivery (VD) [18–20], while infants born by cesarean section (CS) are suggested to be initially colonized by bacteria from the environment such as from maternal skin, hospital staff or other neonates [21].

The first weeks of life is a period in which the intestinal microbiota is very dynamic, with nutrition governing the developing ecosystem [15]. Breastfed infants typically have a bifidobacteria-dominated microbiota whereas formula-fed infants have a more diverse microbiota. After the introduction of solid foods, bacterial succession continues gradually diversifying with adult-like species such as *Bacteroides* spp. and *Clostridium* cluster IV and XIV. The exact age at which a stable adult-like composition is established is still unclear but it is thought to be around 3 years of age [22, 23]. However, this process continues past 3 years of age, and events occurring later in life, such as hormonal changes during puberty or changes in eating habits may also influence the microbiota composition [24, 25].

To what extend early colonization is influencing the microbiota composition in later life needs to be further elucidated. However, increasing evidence suggests that the initial colonization does influence gut maturation, immune, brain and metabolic development. Early-life events (ie., mode of delivery, type of feeding, antibiotic use) that are known to influence this process, may thus drive predisposition to diseases later in life [13, 26–31]. It is therefore critical to understand the early colonization process in great detail, including the confounding factors in early life that can be of long term importance.

This study aims to describe the dynamics of early colonization during the first six months of life and identify factors that can drive changes in the composition of the gut microbiota in early life.

## Material and Methods

### Subjects and sampling

The present study was an observational study performed in the area of Antwerp (Belgium) between June 2009 and July 2010. The protocol was approved by the ethics committee of the hospital network of Antwerp (ZNA), Institutional Review Board-ZNA/OCMW Antwerpen. The trial is registered in the ISRCTN register: ISRCTN66704989.

One hundred and forty-three healthy pregnant women were recruited via gynecologists. Subjects were eligible for participation when they showed a normal course of pregnancy and a good physical and mental health status as determined by the medical history and general clinical examination according to the investigators’ judgment. Exclusion criteria were 1) Birth in water; 2) Participation in another clinical trial; 3) Alcohol consumption (>7 units/week); 4) Illegal drug use. Written informed consent was obtained before inclusion in the study. Subjects that fulfilled one or more of the following criteria during the study, were excluded from further participation: 1) premature delivery (before 37 weeks of pregnancy); 2) bacterial/viral infection within 2 weeks prior to delivery; 3) congenital malformation(s) in the subjects’ infant; 4) use of immune-modulatory drugs by subject and/or subjects’ infant prior to (4 weeks) delivery and during the study; 5) use of antibiotics between 2 weeks prior to delivery and 2 weeks after delivery, for any reason except for prophylactic use (e.g. cesarean section).

Upon enrolment (about 1–2 months before the expected delivery date) information with respect to the subjects’ demographics, relevant medical history, medication and nutritional supplement use were documented. Subjects were issued with questionnaires to register information on the diet, possible allergic/gastrointestinal symptoms, and medication to be completed at several time points during the study (before delivery, immediately after delivery, at 1/2/3/4 weeks after delivery, and at 2/3/4/5/6 months after delivery).

After delivery fecal samples of the neonates were collected at several time points (first defecation, 2 days after first defecation, 1 week after birth, 1/3/6 months after birth, 1 week after weaning started).

Participants were given a pre-weighed 15 ml fecal collection tube (Sarstedt AG & Co., Numbrecht, Germany) containing 2 ml of RNA*later* (Thermo Fisher Scientific Inc., Waltham, MA), and an empty fecal collection tube. They placed a spoonful of fecal sample (approximately 0.5 g) into each collection tube immediately after defecation. The samples were kept at 4°C in a cooling box with refrigerants and sent to Yakult Honsha European Research Center for Microbiology ESV (Ghent, Belgium), within 72 h of collection.

### Primary treatment of fecal samples

For the following total DNA extraction and 4’,6-diamidino-2-phenylindole (DAPI) staining, fecal samples, which were collected in empty tubes, were suspended in nine volumes of phosphate-buffered saline (PBS) (Nissui Pharmaceutical Co. Ltd., Tokyo, Japan). The fecal suspension in the PBS was then stored at -30°C until DNA extraction.

Another portion of the fecal samples, also from the samples collected in the same empty fecal collection tubes, was suspended in nine volumes of 0.1 M phosphate buffer (pH 5.5) to make a 10-fold fecal suspension for organic acid analysis. Then, 0.8 ml of this fecal suspension, 0.1 ml of 10% (v/v) perchloric acid and 0.1 ml of 100 mM crotonic acid (internal standard) were mixed, and kept at 4°C overnight to remove proteins. After centrifugation at 12,000 × g for 5 min, the supernatant was filtered with 0.45 μm filter and stored at -30°C until organic acid analysis was performed.

For total RNA extraction, the fecal collection tubes, which were included 2 ml of RNA*later* and fecal samples collected, were weighed, and were added fresh RNA*later* to make a fecal suspension (100 mg feces/ml). Then, 0.2 ml of this fecal suspension was added to 1 ml of PBS. After centrifugation of the mixture at 12,000 × g for 5 min, the supernatant was discarded by decantation, and the pellet was stored at -80°C until RNA extraction was performed.

### Determination of bacterial count by DAPI staining

Total bacterial cell counts were determined by DAPI staining, according to a method described by Matsuda *et al*. [32]. Briefly, 100 μl of 10-fold diluted fecal homogenate in PBS was added to 3 volumes of 4% paraformaldehyde-PBS (PFA) solution, which was then incubated at 4°C for 16 h. PFA-treated samples were smeared over a Micro slide glass (Matsunami, Osaka, Japan), stained with Vectashield mounting medium with DAPI (Vector microscope Leica DM6000, image acquisition software QFluoro, and a cooled black-and-white charge-coupled device [CCD] camera Leica DFC350FX; Leica Microsystems, Wetzlar, Germany). The obtained fluorescent particles were analyzed using Image-Pro plus software version 4.5 (Media Cybernetics, Rockville, MD), to count the fluorescent cells in each fecal samples. The bacterial count was expressed as the mean value of 24 fields for each smear sample.

### DNA extraction and qPCR

DNA was extracted from the PBS-suspension mentioned above and subjected to qPCR, according to a method described by Matsuki *et al*. [33]. Briefly, the thawed sample was mixed with 250 μl of extraction buffer (100 mM Tris-HCl, 40 mM EDTA; pH 9.0) and 50 μl of 10% sodium dodecyl sulfate. 300 mg of glass beads (diameter, 0.1 mm) (BioSpec Products, Bartlesville, OK) and 500 μl of TE-saturated phenol (Sigma-Aldrich, St. Louis, MO) were added to the suspension, and the mixture was vortexed vigorously for 30 s using a FastPrep-24 (M.P. Biomedicals, Santa Ana, CA) at a power level of 5.0. Phenol–chloroform extractions were performed, and 250 μl of the supernatant was subjected to isopropanol precipitation. Finally, the DNA was suspended in 1 ml of TE buffer (10 mM Tris-HCl, 1 mM EDTA, pH 8.0).

To determine the population sizes and prevalence of the predominant and subdominant bacterial groups, we used primers specific for the groups and species show in Table 1. PCR amplification and detection were performed with an ABI PRISM 7900HT Sequence Detection System and SDS software (ver 2.3) (Thermo Fisher Scientific). The reaction mixture (10 μl) was composed of 10 mM Tris-HCl, pH 8.3; 50 mM KCl; 1.5 mM MgCl_2_; 200 μM of each dNTP; 1:75,000 dilution of SYBR Green I (Thermo Fisher Scientific); 11 ng/μl of TaqStart antibody (ClonTech, Palo Alto, CA); 0.05 U/μl of Taq DNA polymerase (Takara Bio, Shiga, Japan); 0.25 μM of each of the specific primers; and 1 μl of ×10, ×100, or ×1000 diluted template DNA. The amplification program consisted of one cycle at 94°C for 5 min; 40 cycles at 94°C for 20 s, 50°C or 55°C for 20 s (Table 1) [34], and 72°C for 50 s; and finally one cycle at 94°C for 15 s. Fluorescent products were verified at the last step of each cycle.

**Table 1:** 16S rRNA gene targeted primers and standard strains used for qPCR.

Target	Primer	Sequence (5’-3’)	Annealing Temp (°C)	Reference
*Clostridium coccoides* group	g-Ccoc-F	AAATGACGGTACCTGACTAA	55	[34]
	g-Ccoc-R	CTTTGAGTTTCATTCTTGCGAA		
*Clostridium leptum* group	sg-Clept-F	GCACAAGCAGTGGAGT	55	[35]
	sg-Clept-R	CTTCCTCCGTTTTGTCAA		
*Bacteroides fragilis* group	g-Bfra-F2	AYAGCCTTTCGAAAGRAAGAT	50	[34]
	g-Bfra-R	CCAGTATCAACTGCAATTTTA		
Genus *Bifidobacterium*	g-Bifid-F	CTCCTGGAAACGGGTGG	55	[34]
	g-Bifid-R	GGTGTTCTTCCCGATATCTACA		
*Atopobium* cluster	c-Atopo-F	GGGTTGAGAGACCGACC	55	[35]
	c-Atopo-R	CGGRGCTTCTTCTGCAGG		
Genus *Prevotella*	g-Prevo-F	CACRGTAAACGATGGATGCC	55	[34]
	g-Prevo-R	GGTCGGGTTGCAGACC		
*Bacteroides caccae*	s-Bcac122-F	ACGTATCCAACCTACCTCA	55	[36]
	s-Bcac586-R	ACAACTGACTTAACAATCCG		
*Bacteroides eggerthii*	s-Begg187-F	GTTTTTCCGCATGGTTTCAC	55	[36]
	s-Begg590-R	CTTTCACAACTGACTTAAGCA		
*Bacteroides fragilis*	s-Bfra186-R	AATGATTCCGCATGGTTTCA	55	[36]
	s-Bfra592-R	CAAACTTTCACAACTGACTTAC		
*Bacteroides ovatus*	s-Bova175-F	CCGGATAGCATACGAAYAT	55	[36]
	s-Bova587-R	CACAACTGACTTAACAATCC		
*Bacteroides thetaiotaomicron*	s-Bthe175-F	CCCGATGGTATAATCAGAC	55	[36]
	s-Bthe587-R	CACAACTGACTTAACTGTCC		
*Bacteroides uniformis*	s-Buni188-F	TTCTTCCGCATGGTAGAAC	55	[36]
	s-Buni590-R	CTTTCACAACTGACTTAAGCG		
*Bacteroides vulgatus*	s-Bvul129-F	AACCTGCCGTCTACTCTT	55	[36]
	s-Bvul585-R	CAACTGACTTAAACATCCAT		
*Bifidobacterium adolescentis* group^1^	BiADOg-1a	CTCCAGTTGGATGCATGTC	55	[33]
	BiADOg-1b	TCCAGTTGACCGCATGGT		
	BiADO-2	CGAAGGCTTGCTCCCAGT		
*Bifidobacterium animalis* subsp. *lactis*	Bflact2	GTGGAGACACGGTTTCCC	55	[37]
	Bflact5	CACACCACACAATCCAATAC		
*Bifidobacterium bifidum*	BiBIF-1	CCACATGATCGCATGTGATTG	55	[38]
	BiBIF-2	CCGAAGGCTTGCTCCCAAA		
*Bifidobacterium breve*	BiBRE-1	CCGGATGCTCCATCACAC	55	[38]
	BiBRE-2	ACAAAGTGCCTTGCTCCCT		
*Bifidobacterium catenulatum* group^2^	BiCATg-1	CGGATGCTCCGACTCCT	55	[38]
	BiCATg-2	CGAAGGCTTGCTCCCGAT		
*Bifidobacterium dentium*	BiDEN-1	ATCCCGGGGGTTCGCCT	55	[39]
	BiDEN-2	GAAGGGCTTGCTCCCGA		
*Bifidobacterium longum* subsp. *infantis*	BiINF-1	TTCCAGTTGATCGCATGGTC	55	[39]
	BiINF-2	GGAAACCCCATCTCTGGGAT		
*Bifidobacterium longum* subsp. *longum*	BiLON-1	TTCCAGTTGATCGCATGGTC	55	[39]
	BiLON-2	GGGAAGCCGTATCTCTACG		

^1^The *B*. *adolescentis* group consists of *B*. *adolescentis* genotypes A and B.

^2^The *B*. *catenulatum* group consists of *B*. *catenulatum* and *B*. *pseudocatenulatum*.

### RNA extraction and RT-qPCR

Total RNA fractions were extracted from fecal samples by phenol-chloroform and RT-qPCR was performed, according to a method described by Matsuda *et al*. [32]. DNA’se treatment was not used because previous reports have shown that untreated and DNA’se-treated samples showed identical results, indicating that contaminating DNA does not affect RT-qPCR quantification [32].

RT-qPCR was conducted in a one-step reaction, using a QIAGEN OneStep RT-PCR Kit (QIAGEN, Venlo, The Netherlands) using 10 μl of reaction mixture containing 5 μl of template RNA and each specific primer (Table 2) at a concentration of 0.6 μM. The reaction mixture was dispensed into 384-well optical plates by using a MICROLAB STARlet Liquid Handling Workstation (Hamilton Robotics, Reno, NV). The reaction mixture was incubated at 50°C for 30 min for reverse transcription. The continuous amplification program consisted of one cycle at 95°C for 15 min and 40 or 45 cycles at 94°C for 20 s, 55°C or 60°C for 20 s, and 72°C for 50 s. Amplification and detection were performed by using an ABI PRISM^®^ 7900HT Sequence Detection System (Thermo Fisher Scientific).

**Table 2:** 16S or 23S rRNA gene targeted primers and standard strains used for RT-qPCR.

Target	Primer	Sequence (5’-3’)	PCR Cycle N	Annealing Temp (°C)	Ref
*Clostridium perfringens*	s-Clper-F	GGGGGTTTCAACACCTCC	40	60	[32]
	ClPER-R	GCAAGGGATGTCAAGTGT			[40]
Family *Enterobacteriaceae*	En-Isu-3F^1^	TGCCGTAACTTCGGGAGAAGGCA	40	60	[41]
	En-Isu-3R^1^	TCAAGGCTCAATGTTCAGTGTC			
Genus *Enterococcus*	g-Encoc-F	ATCAGAGGGGGATAACACTT	40	55	[32]
	g-Encoc-R	ACTCTCATCCTTGTTCTTCTC			
Genus *Staphylococcus*	g-Staph-F	TTTGGGCTACACACGTGCTACAATGGACAA	40	60	[32]
	g-Staph-R	AACAACTTTATGGGATTTGCWTGA			
*Lactobacillus fermentum*	LFer-1	CCTGATTGATTTTGGTCGCCAAC	40	55	[32]
	LFer-2	ACGTATGAACAGTTACTCTCATACGT			
*Lactococcus lactis*	sg-Lclac-F	TGTAGGGAGCTATAAGTTCTCTGTA	40	60	[42]
	sg-Lclac-R	GGCAACCTACTTYGGGTACTCCC			
*Lactobacillus casei* subgroup	sg-Lcas-F	ACCGCATGGTTCTTGGC	40	60	[32]
	sg-Lcas-R	CCGACAACAGTTACTCTGCC			
*Lactobacillus gasseri* subgroup	sg-Lgas-F	GATGCATAGCCGAGTTGAGAGACTGAT	40	60	[32]
	sg-Lgas-R	TAAAGGCCAGTTACTACCTCTATCC			
*Lactobacillus plantarum* subgroup	sg-Lpla-F	CTCTGGTATTGATTGGTGCTTGCAT	40	60	[32]
	sg-Lpla-R	GTTCGCCACTCACTCAAATGTAAA			
*Lactobacillus sakei* subgroup	sg-Lsak-F	CATAAAACCTAMCACCGCATGG	45	60	[32]
	sg-Lsak-R	TCAGTTACTATCAGATACRTTCTTCTC			
*Lactobacillus reuteri* subgroup	sg-Lreu-F	GAACGCAYTGGCCCAA	45	60	[32]
	sg-Lreu-R	TCCATTGTGGCCGATCAGT			
*Lactobacillus ruminis* subgroup	sg-Lrum-F	CACCGAATGCTTGCAYTCACC	40	60	[32]
	sg-Lrum-R	GCCGCGGGTCCATCCAAAA			

^1^Specific primers are targeting 16S rRNA genes, except for En-Isu-3F/En-Isu-3’R, which targets 23S rRNA genes

### Determination of bacterial number by qPCR and RT-qPCR

The bacterial counts was estimated from the slope of the standard curve as described earlier [43], generated by using the following standard strains: *Ruminococcus productus* ATCC 27340^T^ (for the *Clostridium coccoides* group), *Faecalibacterium prausnitzii* ATCC 27768^T^ (for the *Clostridium leptum* group), *Bacteroides vulgatus* ATCC 8482^T^ (for the *Bacteroides fragilis* group), *Bifidobacterium longum* subsp. *longum* ATCC 15707^T^ (for the Genus *Bifidobacterium*), *Collinsella aerofaciens* ATCC 25986^T^ (for the *Atopobium* cluster), *Prevotella melaninogenica* ATCC 25845^T^ (for the Genus *Prevotella*), *Bacteroides caccae* ATCC 43185^T^ (for *Bacteroides caccae*), *Bacteroides eggerthii* ATCC 27754^T^ (for *Bacteroides eggerthii*), *Bacteroides fragilis* ATCC 25285^T^ (for *Bacteroides fragilis*), *Bacteroides ovatus* ATCC 8483^T^ (for *Bacteroides ovatus*), *Bacteroides thetaiotaomicron* ATCC 29148^T^ (for *Bacteroides thetaiotaomicron*), *Bacteroides uniformis* ATCC 8492^T^ (for *Bacteroides uniformis*), *Bacteroides vulgatus* ATCC 8482^T^ (for *Bacteroides vulgatus*), *Bifidobacterium adolescentis* ATCC 15703^T^ (for the *Bifidobacterium adolescentis* group), *Bifidobacterium animalis* subsp. *lactis* DSM 10140^T^ (for *Bifidobacterium animalis* subsp. *lactis*), *Bifidobacterium bifidum* ATCC 29521^T^ (for *Bifidobacterium bifidum*), *Bifidobacterium breve* ATCC 15700^T^ (for *Bifidobacterium breve*), *Bifidobacterium pseudocatenulatum* JCM 1200^T^ (for the *Bifidobacterium catenulatum* group), *Bifidobacterium dentium* ATCC 27534^T^ (for *Bifidobacterium dentium*), *Bifidobacterium longum* subsp. *infantis* ATCC 15697^T^ (for *Bifidobacterium longum* subsp. *infantis*), *Bifidobacterium longum* subsp. *longum* ATCC 15707^T^ (for *Bifidobacterium longum* subsp. *longum*), *Clostridium perfringens* JCM 1290^T^ (for *Clostridium perfringens*), *Escherichia coli* ATCC 11775^T^ (for the Family *Enterobacteriaceae*), *Enterococcus faecalis* ATCC 19433^T^ (for the Genus *Enterococcus*), *Staphylococcus aureus* ATCC 12600^T^ (for the Genus *Staphylococcus*), *Lactobacillus fermentum* ATCC 14931^T^ (for *Lactobacillus fermentum*), *Lactococcus lactis* subsp. *lactis* JCM 5805^T^ (for the *Lactococcus lactis* subgroup), *Lactobacillus casei* ATCC 334^T^ (for the *Lactobacillus casei* subgroup), *Lactobacillus gasseri* DSM 20243^T^ (for the *Lactobacillus gasseri* subgroup), *Lactobacillus plantarum* ATCC 14917^T^ (for the *Lactobacillus plantarum* subgroup), *Lactobacillus sakei* subsp. *sakei* JCM 1157^T^ (for the *Lactobacillus sakei* subgroup), *Lactobacillus reuteri* JCM 1112^T^ (for the *Lactobacillus reuteri* subgroup), and *Lactobacillus ruminis* JCM 1152^T^ (for the *Lactobacillus ruminis* subgroup).

### Identification and quantification of fecal organic acids

The supernatant, which was prepared as mentioned in the section entitled Primary treatment of fecal samples, was injected into a Water 432 Conductivity Detector (Waters Corporation, Milford, MA) to determine the concentrations of fecal organic acids (acetic acid, butyric acid, formic acid, iso-valeric acid, lactic acid, propionic acid, succinic acid, and valeric acid) by HPLC as previously reported [44].

### Statistical Analyses

The data of bacterial counts contained proportions of non-detected values due to detection limits. The non-detected values were substituted with the detection limit divided by the square root of 2 when the percentage of non-detected samples at a time point was less than 10% [45]. When the percentage of non-detected samples at a time point was greater than 40% then the outcome was directly converted to a binary result (detected versus non-detected). The remaining outcomes that had a percentage of non-detected samples between 10% and 40% at a time point were considered for imputation. At each time point a semi-parametric density estimate was fitted to the bacterial counts and the non-detected values were replaced by imputed values from this estimated semi parametric density [46].

Analysis by random coefficients mixed model (for continuous outcome variables) and generalized linear mixed model (for binary outcome variables) were used to study associations between the outcome variables (gut microbiota and organic acids) and potential covariates. The effect of mode of delivery, type of feeding, antibiotic use, number of siblings, presence of allergens, and gender on colonization of the gut was studied. The selection of important covariates was done using likelihood ratio tests, starting from full models including all potential covariates and where possible covariate by time interaction terms. The covariate selection continued using a backward selection method. At each step, changes in the standard errors and point estimates were checked in order not to miss any confounding effects.

First defecation samples (meconium) were analyzed separately from the samples taken at other time points due to high percentages of specific assays being below detection limit in these samples. Time (in days) at which first defecation sample was taken was included as an additional covariate.

The measured gut microbiota variables and organic acids values from samples taken at 2, 7, 30 days and 3 months were analyzed in a longitudinal model that started with the assumption of a second order polynomial trajectory over time. However, during the backward selection, the time-squared terms could be deleted from the model if there was no suggestion for a curvature over time.

Since solid foods were introduced from 4 months onwards, the six months samples were analyzed separately and this variable was included as an additional covariate.

In order to check the consistency of our modeling results with the observed data, we included figures of the observed data and checked them against figures obtained from the fitted models. For the species that were not detected in at least 40% of the samples, we used the binary variable as an outcome (detected versus non-detected), permitting us to obtain the probability to be detected from the fitted models (with the correspondent p-value) which can be examined with the prevalence calculated from the observed data. For the species that were detected in at least 60% of the samples, we used the continuous variable as an outcome (log10 cell count/g of feces or μmol/g of feces for bacteria or organic acids, respectively), thus we could obtain the estimated amount from the fitted model (with the correspondent p-value) and the observed data as measured.

## Results & Discussion

### Study population

143 subjects were included in the study of which 34 dropped-out during the study. No longer fulfilling the inclusion and exclusion criteria during the post-delivery re-check, was considered the major reason for drop out (N = 18). The other early terminators were due to withdrawal of informed consent (N = 4), protocol violation (N = 6), lost to follow up (N = 2), and for other reasons (N = 4). 109 subjects were included in the study but data of 3 subjects was not properly collected and two subjects delivered twins, so in total fecal samples of 108 subjects were suitable to be analyzed. The study population did not have a significant difference in the numbers of boys and girls (51.9 and 48.1%, respectively), and half of the total population had at least one older sibling (48.1%). Twenty eight infants were born by CS (25.9%) and only 5 out of the 108 infants were born at home (4.6%).

Other characteristics that vary in time are summarized in Table 3. These characteristics include type of feeding (breastfeeding, mix feeding or formula feeding at the sampling time), time of introduction of solid food and antibiotic use.

**Table 3:** Population characteristics varying in time.

Characteristics (Number of subjects)		Meconium^1^	+ 2 days^2^	Day 7	Day 30	Day 90	Day 180
Type of feeding	Exclusive breastfeeding	100	76	89	79	59	10
	Exclusive Formula feeding	3	2	3	5	15	0
	Mix feeding^3^	2	4	11	23	30	3
	Introduction of solid food^4^	0	0	0	3	3	88
	Missing information	3	26	5	1	1	7
Antibiotic use	Yes/no	0/108	0/108	0/108	0/108	2/106	6/102
	Number of eecal samples collected per time point	96	80	98	106	105	102
	Nmber of infants	108	108	108	108	108	108

^1^Meconimum samples were taken at 0.77 ± 1.04 days (mean±st dev)

^2^ The consequent sample was taken 2 days after, which occurred at 2.88 ± 0.99 days (mean±st dev)

^3^Mix-feeding is defined as the combination of breastfeeding and formula feeding

^4^ Introduction of solid food does not exclude breastfeeding or mix-feeding.

### Early colonization

It was always believed that the gut of a newborn is sterile and that the colonization process only started at birth. This concept has been challenged in the last years due to the recent discovery of bacteria or bacterial DNA in the umbilical cord blood, placenta and meconium [8–11], suggesting *in utero* exposure to bacteria. In this study, the first fecal sample (meconium) was collected from 96 infants (88.89%) and bacteria were detected in 74 out of the 96 meconium samples (77.08%). The most prevalent bacteria were *Staphylococcus* which was detected in 67.71% of the samples, followed by *Enterobacteriaceae*, *Enterococcus*, *Lactobacillus* and *Bifidobacterium*. The later showed the highest cell count number in meconium (7.69±1.13 log10 cells/g of feces). Interestingly, bacteria were detected in the meconium of infants born by C-section (as described later in this article), suggesting that colonization by certain bacteria might start before birth. Acetic acid was the organic acid the most often detected (63% of the meconium samples) with a mean amount of 9.12 ±8.74 μmol/g of feces.

These results are in agreement with a recent study reporting evidence of the presence of the same spectrum of bacteria in 66% of meconium samples collected within 24 hours after birth [47].

In that study, Hansen *et al*., concluded that there is limited bacterial colonization at birth and it occurs rapidly thereafter. In our study we defined meconium as the first fecal sample after birth but collection was not restricted to the first 24 hours of birth, the mean time at which they were collected was at 0.77 days (± 1.04 days). Since time was included in our model as a variable, our results showed that delaying the collection of meconium samples by one day increased the chance of detecting the following bacteria: *Bifidobacterium*; *Bacteroides fragilis* group*; Enterococcus; Staphylococcus; L*. *gasseri* subgroup; and *B*. *adolescentis* by 3.1 (p = 0.003); 2.35 (p = 0.01); 10.69 (p<0.001); 2.26 (p = 0.016), 3.01 (p = 0.018); and 3.9 (p = 0.008) fold respectively. This could be explained by the growth of microorganisms that were already present before delivery as recently suggested [8–11] or by the presence of those microorganisms entering the ecosystem from the environment after birth.

After 2 days, the prevalence and count of the different bacterial groups followed a temporal and dynamic change during the first 6 months of life as illustrated in S1 Table.

The microbiota composition evolves over time which is the result of the combined impact of different events (i.e. different type of feeding, mode of delivery, and antibiotic use) and will be discussed below. Interestingly, for some species no suggestion for a statistically significant influence of any of the covariates emerged except for time. Examples are the increased probability to detect *B*. *uniformi*s during the first 3 months of life (longitudinal model, p = 0.003), the decreased probability to detect *L*. *fermentum* (six months samples; p = 0.049), the increased probability to detect *B*. *animalis* subsp. *lactis* (six months samples; p = 0.0046 and the decreased in *Staphylococcus* counts from 3 to 6 months (six months samples; p = 0.009). These findings may suggest that these bacteria are transient in the infant gut but may play a key role in maturing the immune system in the first months of life.

### Influence of mode of delivery on the infant gut microbiota during the first 6 months of life

The mode of delivery is often described as a key factor in selecting the early colonizers of the infant gut. Infants born by VD are exposed to a vast number of bacteria when passing through the birth canal, whereas CS-born infants lack the direct contact with the maternal microbiota, and are exposed to a more sterile environment at birth. It has been suggested that the aberrant gut microbiota composition observed in early life in infants born by CS may explain why CS-born infants are at a higher risk for developing allergy and obesity later in life [26, 29, 48–51].

In this study (in which 25.9% infants were born by CS) mode of delivery was indeed found to be one of the factors that had a strong impact on the early microbiota composition. Infants born by VD had significantly higher bacterial counts in the meconium (as measured by DAPI) than those born by CS (8.47 vs. 7.76 log10 cells/g of feces, first defecation sample, p = 0.024), which may have resulted from the transfer of bacteria during birth. This difference in total bacteria count was observed during the whole study period, although cell counts increased faster over time in CS-born infants (longitudinal model, p = 0.004). In addition, CS-born infants had a delayed colonization of several bacterial groups or species, and this was still the case for some species at 6 months of age as illustrated in Table 4.

**Table 4:** Prevalence of different bacterial groups in infants born by vaginal delivery or C-section.

Prevalence (%)	*Vaginal Delivery (N = 80)*						*Cesarean section (N = 28)*					
	Mec^1^	D2^2^	D7	D30	D90	D180	Mec^1^	D2^2^	D7	D30	D90	D180
***Bifidobacteria***	16.7	82.3	90.7	94.9	97.4	97.4	16.7	22.2	69.6	75	100	100
***Bacteroides fragilis group***	12.5	80.7	68	74.4	77.9	82.9	4.2	0	0	3.6	32.1	57.7
***Enterococcus***	21.3	62.9	72.6	72.2	89.7	97.4	12	61.1	75	92.9	96.5	100
***Clostridium perfringens***	5.3	11.3	21.9	24.1	33.3	36.4	0	16.7	50	53.6	35.7	34.6
***Atopobium***	1.4	29	29.3	39.7	54.5	71.4	0	0	0	17.9	64.3	69.2
***B*. *fragilis***	2.8	20.9	12.5	12.8	22.1	34.2	0	0	0	3.6	14.3	26.9
***B*. *caccae***	1.4	17.7	5.3	10.3	18.2	23.7	0	0	0	3.6	10.7	11.5
***B*. *vulgatus***	11.11	67.7	56	58.9	66.2	65.8	0	0	0	3.6	14.3	38.5
***B*. *ovatus***	1.4	14.5	17.3	25.6	24.7	30.3	0	0	0	0	7.1	7.7
***B*. *uniformis***	0	22.6	14.7	21.8	31.2	40.8	0	0	0	3.6	14.3	15.4
***B*. *catenulatum* group**	2.8	29	28	29.5	37.7	55.3	0	0	0	7.1	28.6	53.9
***B*. *longum***	11.1	62.9	69.3	71.8	77.92	78.9	0	0	8.7	39.3	71.4	96.2
***B*. *bifidum***	12.5	51.6	45.3	50	66.2	77.6	8.3	11.1	4.3	21.4	60.7	69.2
***L*. *gasseri* subgroup**	17.3	70.1	39.7	49.3	39.7	35.1	8	11.11	33.3	46.4	50	26.9
***L*. *reuteri* subgroup**	20	20.9	17.8	18.9	23.1	22.1	4	5.5	8.3	21.4	21.4	19.2

Table representing only those bacterial groups that were significantly dependent on mode of delivery; Prevalence presented as % of infants in which the bacteria were detected

^1^The meconium sample was taken at 0.77 ± 1.04 days (mean±st dev)

^2^ The consequent sample was taken 2 days after, which occurred at 2.88 ± 0.99 days (mean±st dev)

**Fig 1:**
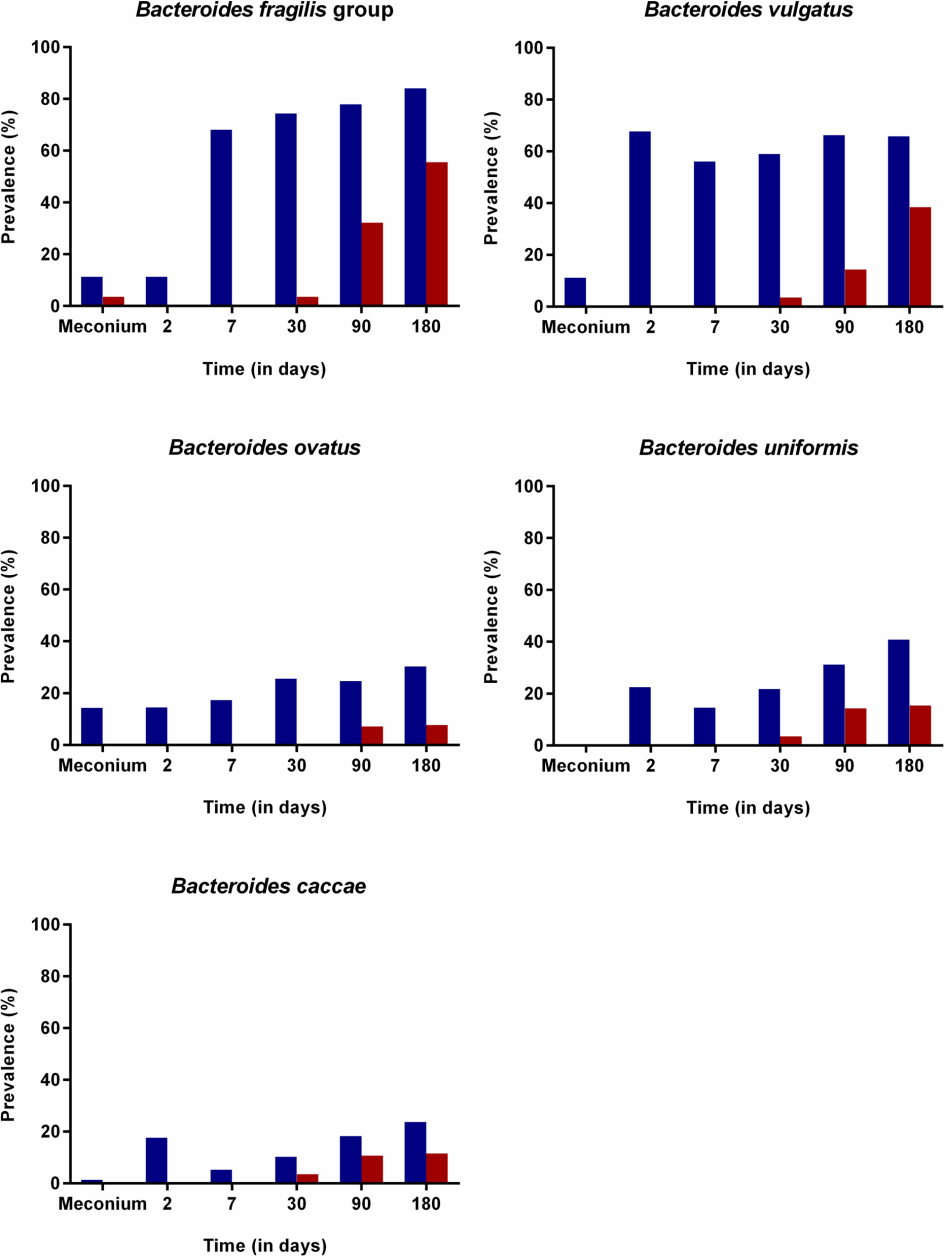
Effect of mode of delivery on the colonization by *Bacteroides* species. Prevalence of different *Bacteroides* species in fecal samples of infants born by caesarean section (red) or vaginal delivery (blue) at birth, 2, 7, 30, 90 and 180 days of life, using the original data.

**Fig 2:**
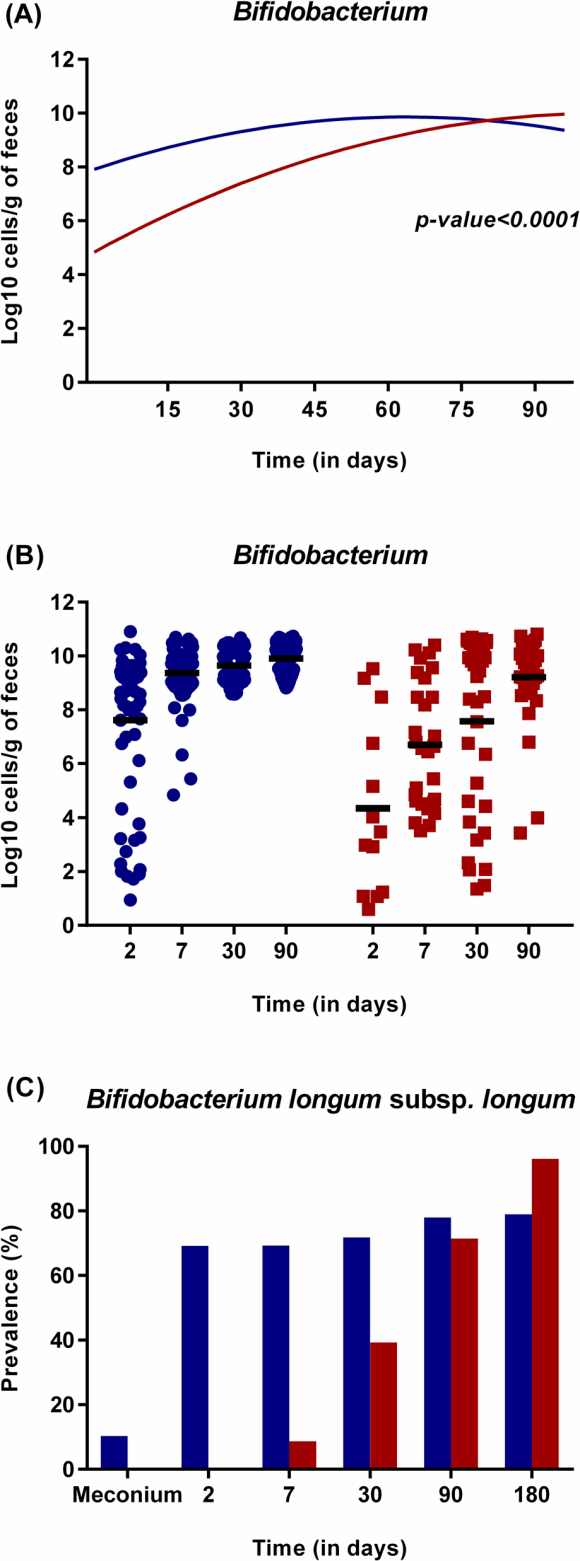
Effect of mode of delivery on the colonization by *Bifidobacterium*. *Bifidobacterium* count (in log10 cell/g of feces) in infants born by caesarean section (red) compared to those born by vaginal delivery (blue) over time as calculated by the statistical model (A) or as represented from original data (B). (C) Prevalence of *B*. *longum* in infants born by CS (red) or vaginal delivery (blue) at birth, 2, 7, 30, 90 and 180 days of life. The graph is generated using the raw data.

**Fig 3:**
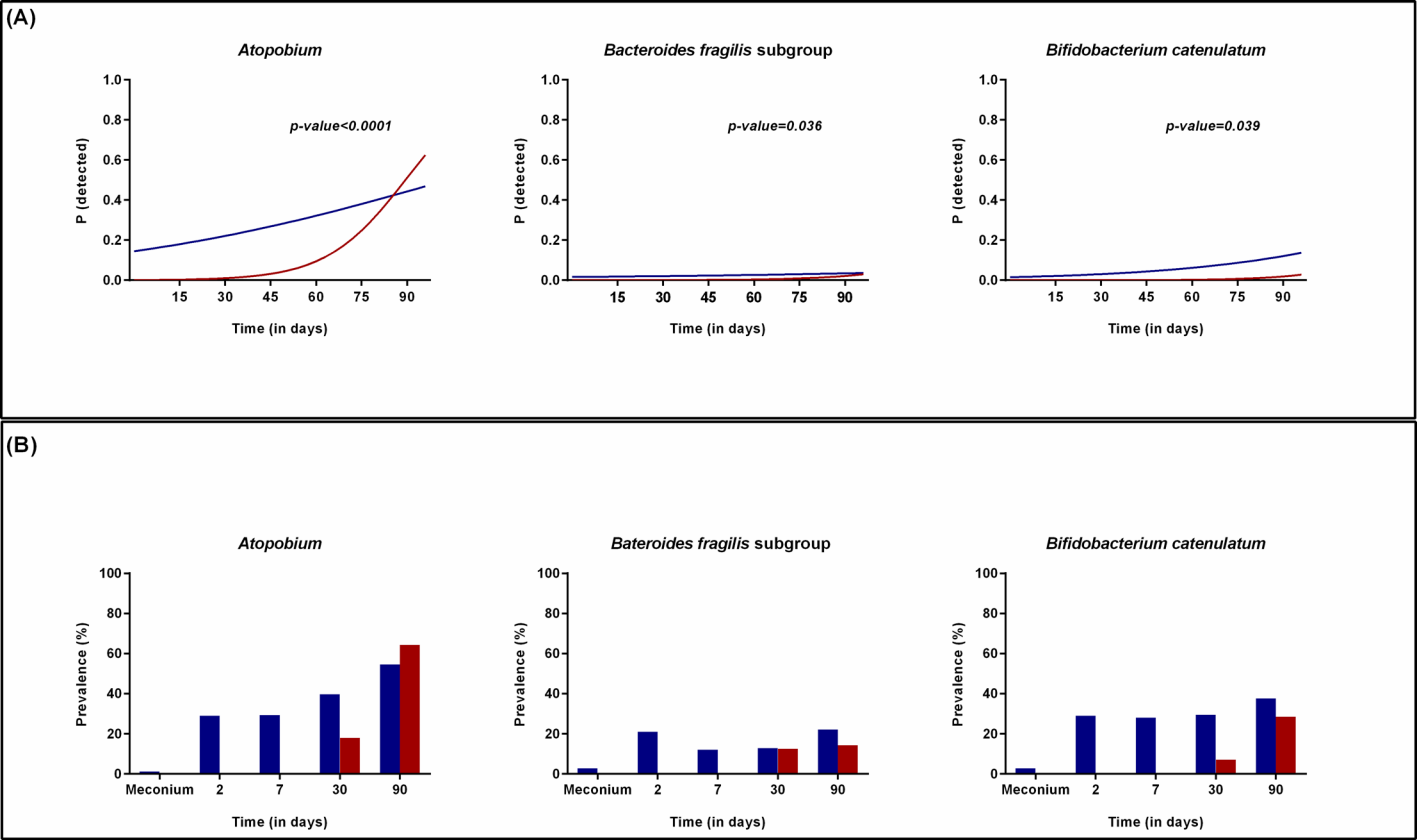
Effect of mode of delivery on the probability to detect different bacteria. (A) Probability to detect *Atopobium* cluster, *B*. *fragilis* group and *B*. *catenulatum* group in infants born by VD (blue) compared to those born by CS (red) over time as calculated by the statistical model. (B) Prevalence of *Atopobium* cluster, *B*. *fragilis* group and *B*. *catenulatum* group in in infants born by VD (blue) compared to those born by CS (red) over time as represented by the original data.

The delayed colonization in CS-born infants was demonstrated by the significantly lower probability to detect *Bacteroides fragilis* group (longitudinal model, p = 0.003), *B*. *ovatus* (longitudinal model, p = 0.006), *B*. *vulgatus* (longitudinal model, p<0.001), *B*. *uniformis* (longitudinal model, p = 0.001), *B*. *caccae* (longitudinal model, p = 0.028) (Original data represented in Fig 1), and *B*. *longum* subsp. *longum* (longitudinal model, p<0.001) (Original data represented in Fig 2) during the first three months of life. During this period, bifidobacterial counts were significantly higher in VD-born infants, although it increased rapidly in the CS group (longitudinal model, p<0.001) (Fig 2A). In addition, the probability to detect *Atopobium* cluster (longitudinal model, p<0.001), *B*. *fragilis* subgroup (longitudinal model, p = 0.036) and *B*. *catenulatum* group (longitudinal model, p = 0.039) in VD-infants was higher than in CS-born infants and followed a different trajectory over time (Fig 3A).

**Fig 4:**
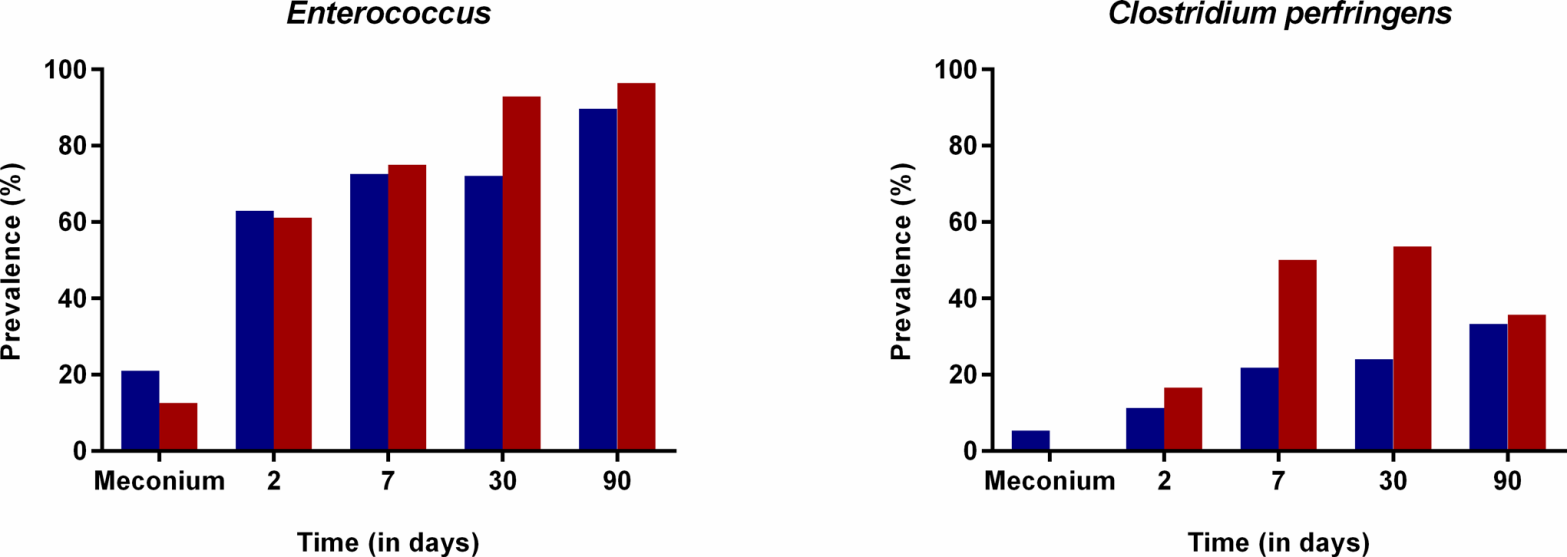
Effect of mode of delivery on the colonization by *Enterococcus* and *Clostridium perfringens*. Prevalence of *Enterococcus* and *Clostridium perfringens* in fecal samples of infants born by caesarean section (red) or vaginal delivery (blue) at birth, 2, 7, 30, 90 and 180 days of life, using the original data.

As previously described, the lack of bacterial transmission during CS delivery or more likely; the exposure to sterile hospital surfaces or the skin of medical personnel/parents, seems to favor the colonization and growth of facultative aerobes and skin related bacteria such as *Staphylococcus*, *Propionibacterium* and *Clostridium* species [52, 53]. In our study, the probability to detect *Enterococcus* (longitudinal model, p = 0.008) and *C*. *perfringens* (longitudinal model, p = 0.013) was significantly higher in infants born by CS compared to those born by VD (Original data represented in Fig 4).

There is no consensus about the timing at which the differences observed between the microbiota composition of VD and CS-born infants may disappear, if at all. Whereas some authors have reported that there are no differences after 1 month, others reported differences even at 7 years of age [52]. In our study most differences disappeared at 6 months of age, except for the higher probability to detect *B*. *fragilis*-group, *B*. *uniformis*, *B*. *vulgatus* and *B*. *ovatus* (six months samples, p = 0.01, p = 0.002, p = 0.007, p = 0.008, respectively) in VD-born than in CS-born infants (Original data represented in Fig 1) for which longer study periods than the 6 months studied here would be required.

**Fig 5:**
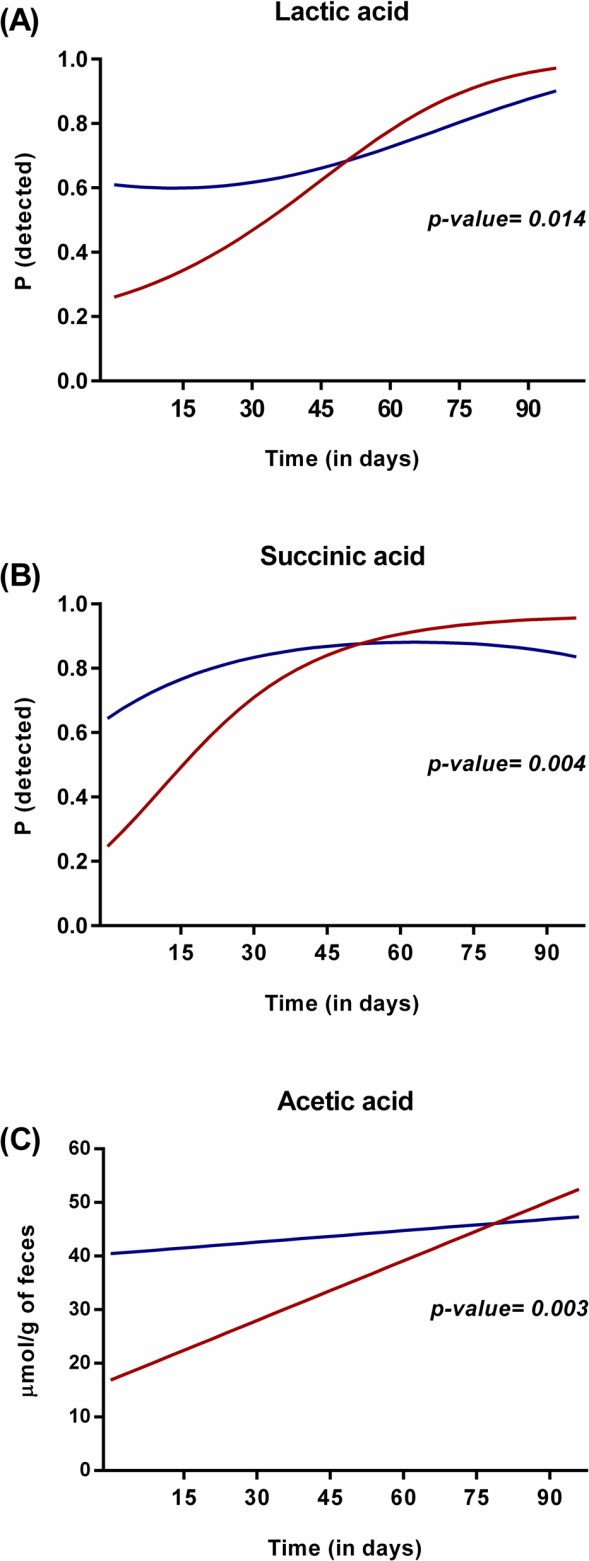
Effect of mode of delivery on the organic acid profile. Probability to detect organic acids (acetic, lactic and succinic acid) in infants born by caesarean section (red) compared to those born by vaginal delivery (blue) over time as calculated by the statistical model.

The differences observed in the microbiota composition of infants born by CS or VD were also in line with differences in organic acids detected in the fecal samples. Significant differences were found for the detection of succinic acid (longitudinal model, p = 0.004) and lactic acid (longitudinal model p = 0.014), and for the amount of acetic acid (longitudinal model p = 0.003) between the two groups (Fig 5). When analyzing the data at each time point separately, succinic and lactic acid were most often detected in infants born by VD only at day 2 (p<0.001 and p = 0.026, respectively) while the amount of acetic acid was significant until 1 week (p = 0.04). The pattern of organic acids described reflects the colonization pattern with high levels of bifidobacteria in these two groups of infants (VD vs CS). The same trajectory over time was observed for the *Bifidobacterium* count and the acetic acid concentration, one of the major metabolites produced by *Bifidobacterium* as a result of the metabolism of carbohydrates (Figs 2A and 5C).

Our results are in agreement with previous studies reporting a delayed colonization by *Atopobium* cluster, *Bifidobacterium* and *Bacteroides* species in infants born by CS, whereas they are more often colonized by *Enterococcus* and *Clostridium* species [12, 15, 54–57]. However, we could not confirm the higher prevalence of *Staphylococcus* in CS-born infants previously described by Dominguez-Bello *et al*. [53], which might be explained by the geographical differences of the study populations (Europe and USA).

Our data confirmed that the mode of delivery influences the early colonization and development of the infant gut microbiota, and showed that differences in species composition can still be found until at least 6 months of age.

#### Impact of type of feeding on the differences observed as a function of mode of delivery

It is often suggested that the differences observed in the microbiota composition of infants born by CS are also due to a different type of feeding. It is even argued that breastfeeding is more difficult after a CS delivery [58, 59], and therefore, that infants born by CS are often not breastfed, or that they start breastfeeding later than infants born by VD. The results we have reported so far were found to be dependent only on the mode of delivery, however, in this study we also found that the presence of the *L*. *reuteri* subgroup, the *L*. *gasseri* subgroup and *B*. *bifidum* was dependent on the interaction between mode of delivery and the type of feeding (longitudinal model; p = 0.003, p = 0.03, p = 0.009, respectively) (Table 5).

**Table 5:** Impact of mode of delivery and type of feeding^1^.

Prevalence		Vaginal delivery		Caesarean section	
Species	Time	Exclusive BF % (N)	Mix Feeding % (N)	Exclusive BF % (N)	Mix Feeding % (N)
*B*. *bifidum*	Meconium^2^	11.26 (71)	25 (4)	8.33 (25)	0 (1)
	Day 2^3^	51.72 (58)	50 (4)	5.58 (17)	50 (2)
	Day 7	47.69 (65)	37.5 (8)	5.58 (17)	0 (6)
	Day 30	51.66 (60)	41.18 (17)	11.76 (17)	36.36 (11)
	Day 90	70.45 (44)	60.60(33)	46.15 (13)	73.33 (15)
*L*. *gasseri*	Meconium^2^	18.3 (71)	0 (4)	8 (25)	0 (1)
	Day 2^3^	55.17 (58)	25 (4)	11.76(17)	0 (2)
	Day 7	41.64 (65)	25 (8)	33.33 (18)	33.33 (6)
	Day 30	47.54 (61)	41.18 (17)	47.06 (17)	45.45 (11)
	Day 90	40 (45)	40.63 (32)	76.92 (13)	23.08(13)
*L*. *reuteri*	Meconium^2^	8.45 (71)	0 (4)	4 (25)	0 (1)
	Day 2^3^	22.41 (58)	0 (4)	5.58 (17)	0 (2)
	Day 7	16.92 (65)	25 (8)	0 (18)	33.33 (6)
	Day 30	18.03 (61)	23.53 (17)	5.88 (17)	45.45 (11)
	Day 90	11.11 (45)	40.63 (32)	15.38 (13)	15.38 (13)

^1^Original data

^2^The meconium sample was taken at 0.77 ± 1.04 days (mean±st dev)

^3^ The consequent sample was taken 2 days after, which occurred at 2.88 ± 0.99 days (mean±st dev)

N (Number of infants born by VD or CS and being exclusive or mix-fed). % (proportion of infants in which the specific species was detected)

Infants born by VD who were exclusively breastfed were the ones most often colonized by *B*. *bifidum* (longitudinal model, p = 0.009) and *L*. *gasseri* subgroup (longitudinal model, p = 0.029), compared to those exposed to formula feeding (mixed feeding) or born by CS.

Numbers of *B*. *bifidum* were higher in infants born by VD during the first week of life. However, infants born by CS, who were exclusively breast fed, reached a level comparable to that observed in VD infants earlier than CS-born infants that were exposed to formula (mixed feeding). Similarly, members of the *L*. *gasseri* subgroup were more frequently detected in exclusively breastfed infants. Interestingly, at 3 months, the highest prevalence of both *L*. *gasseri* subgroup and *B*. *bifidum* was found in infants born by cesarean section. All together, these results suggest that vaginal delivery in combination with breastfeeding favor the colonization by *B*. *bifidum* and *L*. *gasseri* subgroup in the infant gut, and that breastfeeding may compensate in time in those infants born by CS by favoring the colonization of these species. Both bacterial species have been previously found in human milk which may explain the differences observed [60, 61]. On the contrary, infants born by CS and exposed to formula feeding were most often colonized by *L*. *reuteri* subgroup, whereas those born by CS but exclusively breastfed were the least often colonized by this species (Table 5). Our results suggest that exposure to formula feeding seems to favor the colonization of this species which may be the result of the use of formula supplemented with probiotic strains belonging to this species.

To our knowledge this is the first time that an interaction between mode of delivery and type of feeding is reported. However, the number of infants born by CS was low and the findings need to be confirmed in future trials.

### Influence of the type of feeding on the infant gut microbiota during the first 6 months of life

The feeding pattern varied along the study and it is described in Table 3. Most of the infants were exclusively breastfed in the first month of life (73.15%), and thus only a small proportion received infant formula either exclusively or in combination with breastfeeding.

At 3 months of age, 54.63% of the infants were still exclusively breastfed, 13.88% were exclusively formula fed, 27.77% received mix feeding, while 2.77% of the infants had received solid foods. At 6 months of age, 9.26% of infants were exclusively breastfed and 2.94% received mix feeding but did not receive solid foods, whereas 81.48% of the infants had received solid foods even though they might continue receiving breast milk and/or infant formula and are considered within the group of weaned infants (Table 3).

Due to the low numbers of exclusively formula fed infants, the data from these infants and the ones receiving mix-feeding were regarded as one group. The differences reported are found when comparing exclusive breastfeeding to mix feeding (partially breastfed or exclusively formula fed).

During the first 3 months of life, exclusively breastfed infants had lower total bacterial counts (by DAPI staining) than those receiving other type of feeding (longitudinal model, p = 0.027), but had higher counts of *Staphylococcus* (longitudinal model, p = 0.037). *Staphylococcus* has been shown to be transferred from breast milk to the infant gut and is known to be one of the most dominant bacteria found in the infant gut [62]. However, as discussed earlier, it is not yet clear whether it is a permanent or transient colonizer or whether they play a key in role in the infant gut [62].

Infants exposed to formula feeding (either exclusively or in combination with breastfeeding) were more often colonized by *Enterococcus* (longitudinal model, p<0.001), *C*. *coccoides* group (longitudinal model, p = 0.002), *Atopobium* cluster (longitudinal model, p = 0.002), *B*. *vulgatus* (longitudinal model, p = 0.027) and *B*. *longum* subsp. *longum* (longitudinal model, p = 0.033) (Original data represented in S1 Fig).

**Fig 6:**
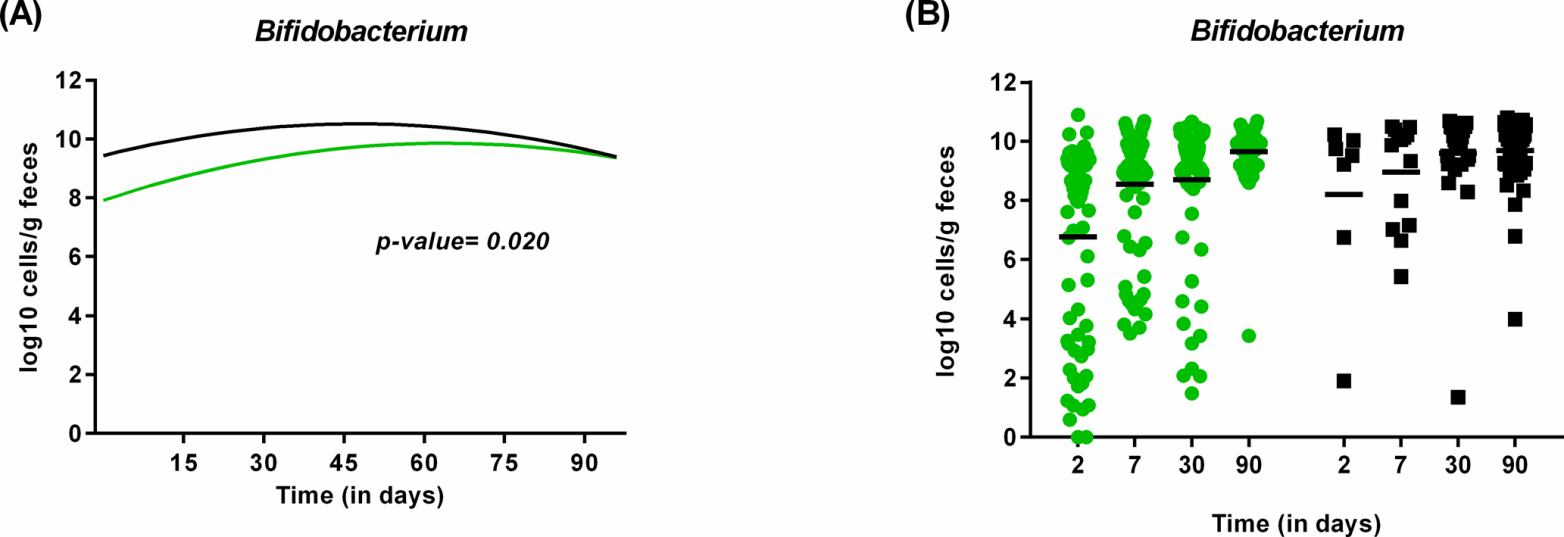
Influence of type of feeding on the colonization by *Bifidobacterium* during the first 3 months of life. *Bifidobacterium* count (in log10 cell/g of feces) in exclusively breastfed infants (green) compared to those receiving mix-feeding (black) over time as calculated by the statistical model (A) or as represented from the original data (B).

**Fig 7:**
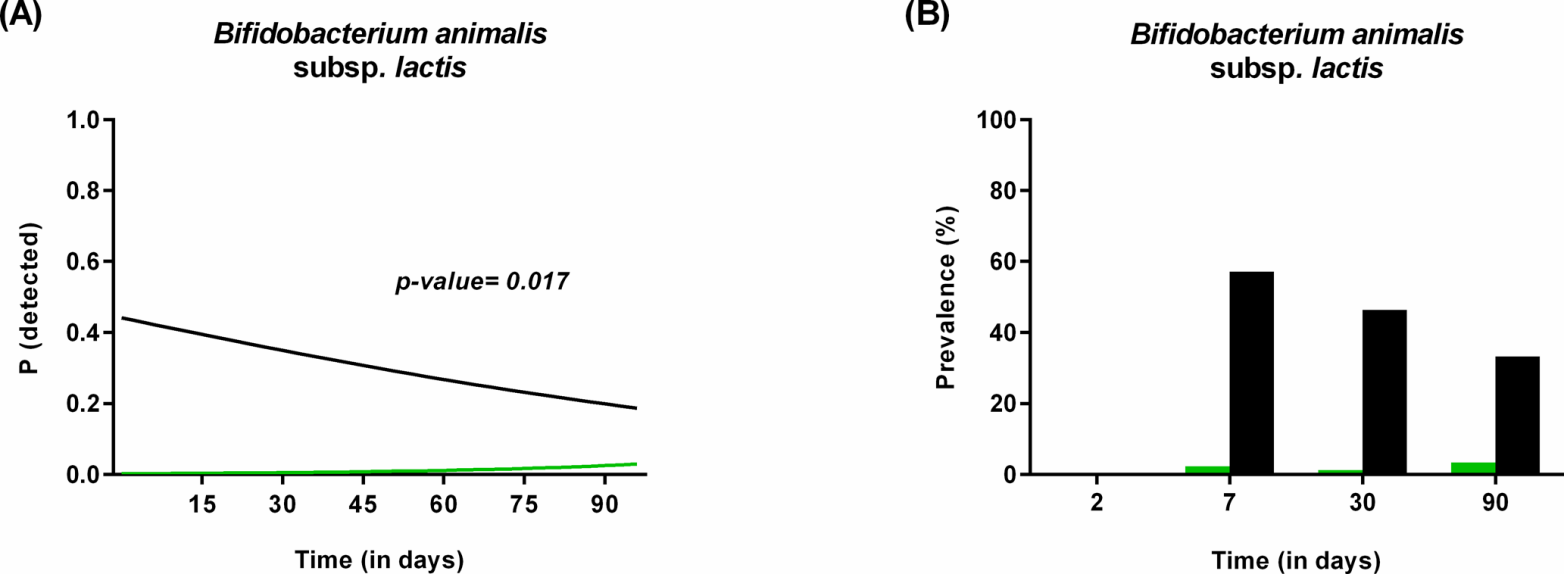
Influence of type of feeding on the presence of *B*. *animalis* subsp. *lactis* during the first 3 months of life. (A) Probability to detect *B*. *animalis* in exclusively breastfed infants (green) compared to those receiving mix-feeding (black) as calculated by the statistical model. (B) Prevalence of *B*. *animalis* in infants receiving mix-feeding (black) or exclusively breast fed (green) at birth, 2, 7, 30 and 90 days of life as represented by the original data.

The colonization of *Bifidobacterium*, *C*. *leptum* group, *C*. *perfringens*, *L*. *casei* subgroup and *B*. *animalis* subsp. *lactis* was also influenced by type of feeding, but the differences followed a different trajectory over time. Contrary to previous studies and much to our surprise [63–66], exclusively breastfed infants had lower counts of *Bifidobacterium*, especially at earlier time points (longitudinal model, p = 0.02; Fig 6A). The high bifidobacterial count found in the mix-fed group can however be explained by the inclusion of a few exclusively formula-fed infants that received a formula supplemented with a probiotic strain (*B*. *animalis* subsp. *lactis*). Indeed, infants exposed to formula feeding were most often colonized by *B*. *animalis* subsp. *lactis* (Fig 7A), whereas this species was always below the detection limit in exclusively breastfed infants. This finding, together with the fact that the probability to detect *B*. *animalis* subsp. *lactis* in mix-fed infants decreased over time (longitudinal model, p = 0.017; Fig 7A), shows that this species is not a common inhabitant of the gut of healthy breast-fed infants.

**Fig 8:**
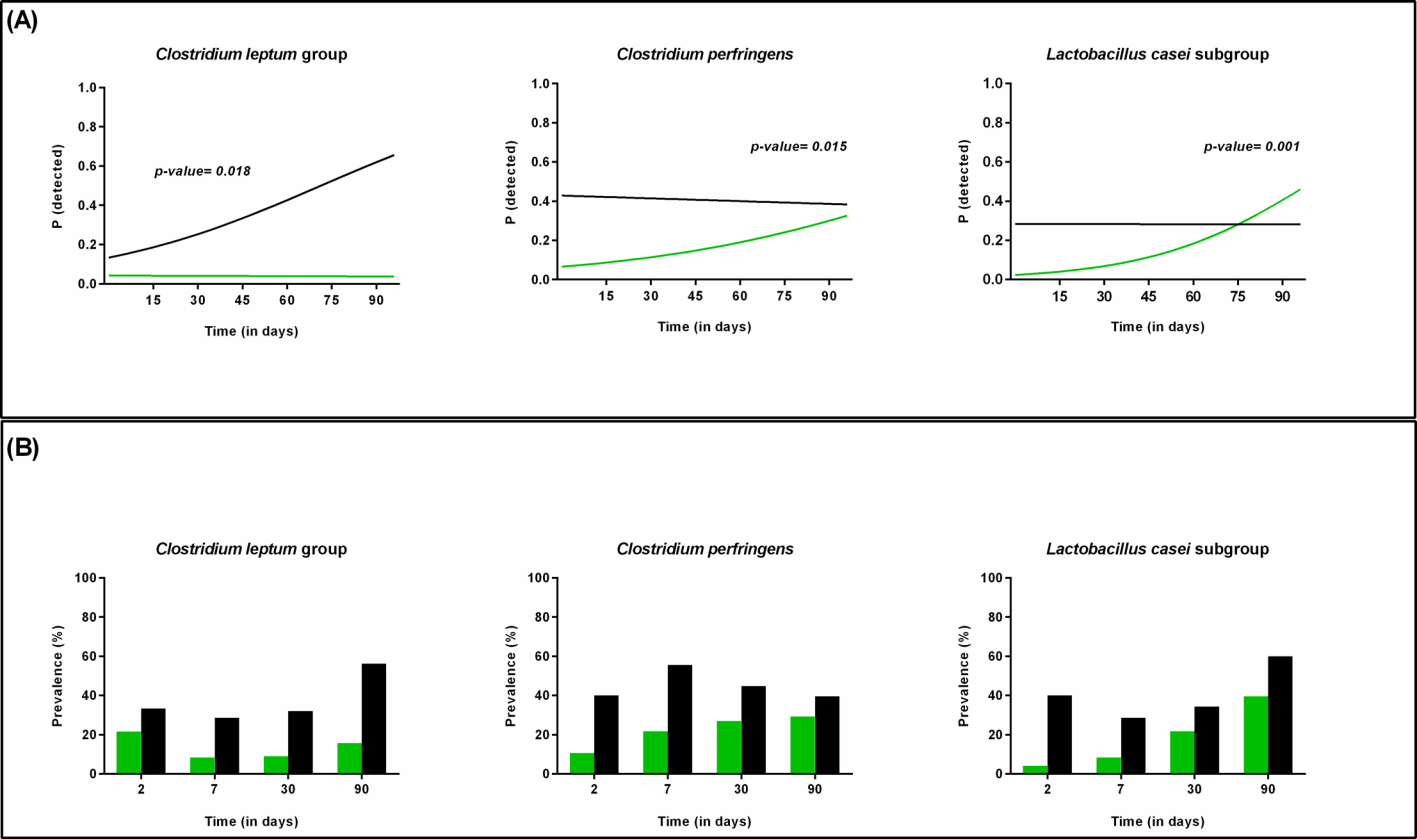
Effect of type of feeding on the colonization pattern of different bacteria during the first 3 months of life. (A) Probability to detect *C*. *leptum* group, *C*. *perfringens*, and *L*. *casei subgroup* in infants receiving exclusive breastfeeding (green) compared to those receiving mix-feeding (black) over time as calculated by the statistical model. (B) Prevalence of *C*. *leptum* group, *C*. *perfringens*, and *L*. *casei subgroup* in infants receiving mix-feeding (black) or exclusive breast feeding (green) over time as represented by the original data.

Exclusively breastfed infants were less often colonized by *C*. *perfringens* (longitudinal model, p = 0.015) and *L*. *casei* subgroup (longitudinal model, p = 0.001), but the probability to detect these bacteria increased more rapidly during the first three months of life in this group of infants than in the group exposed to infant formula (Fig 8). On the other hand, infants exposed to formula feeding were most often colonized by *C*. *leptum* group, and its prevalence increased over time (longitudinal model, p = 0.018; Fig 8).

After 3 months, a considerable number of infants ceased breastfeeding (45%) and/or received solid food. In our study, we investigated the effect of these two factors on the microbiota composition of the fecal sample taken at 6 months.

Diet in the first 3 months of life still has an impact on the microbiota composition at 6 months. Exclusive breastfeeding for the first 3 months in contrast to mix-feeding was associated with a higher count of *Bifidobacterium* (10.07 log10 cells/g feces vs 9.49 log10 cells/g feces; six months samples, p = 0.037) and higher prevalence of *L*. *casei* subgroup (9.5 times more prevalent, six months samples, p = 0.019) and *B*. *adolescentis* group (3 times more prevalent, six months samples, p = 0.044) at 6 months (Original data represented in S2A Fig). The probability to detect *L*. *gasseri* subgroup was also higher in those infants exclusively breastfed for 3 months, although it decreased over time (six months samples, p = 0.017) (Data represented in S3 Fig). The predominance of lactic acid producers described above is also accompanied by a higher probability to detect lactic acid (almost 3 times higher; six months samples, p = 0.049), and lower probability to detect butyric acid (6.4 times lower; six months samples, p = 0.007) (Original data represented in S2B Fig) in those infants exclusively breastfed for 3 months, which may indicate a less complex ecosystem. These results suggest that exclusive breastfeeding in the first three months of life may have a long lasting effect on the colonization process.

Similarly, the introduction of solid foods is associated with the replacement of the early colonizers by a more complex microbiota, which is reflected by a change in the organic acid profile, with lower chance to detect succinic acid (4 times lower, six months samples, p = 0.05) and lactic acid (8 times lower, six months samples, p = 0.015), and a higher chance to detect butyric acid (10 times higher, six months samples, p = 0.05) compared to those infants not receiving solid foods. Indeed, the introduction of solid foods was associated with a higher prevalence of *Atopobium* cluster (six months samples, p = 0.027) and some butyrate-producing bacteria like the *C*. *coccoides* group (six months samples, p = 0.002) (Original data represented in S4 Fig). The increased prevalence of the latter group after the introduction of solid foods has been previously reported, and can be explained by the introduction of complex carbohydrates through the diet that are easily metabolized by these type of bacteria in the infant gut [57, 67, 68]. Another effect of the early introduction of solid food was the decreased amount of early colonizers such as *Enterobacteriaceae* (7.7 log10 cells/g of feces vs. 8.1 log10 cells/g of feces; six months samples, p = 0.04) and *Staphylococcus* (5.06 log10 cells/g of feces vs 5.6 log10 cells/g of feces; six months samples, p = 0.024), which were significantly lower in those infants receiving solid foods than in those not receiving solid foods, as previously described [67]. Unlike other authors, we did not find a decrease in the prevalence of infant-type bifidobacterial species after the introduction of solid foods. Surprisingly, the prevalence of *B*. *longum* subsp. *longum* was even 9.4 times higher (six months samples, p = 0.002). This discrepancy may be due to different ages of the children in the different studies or to the influence that exclusive breastfeeding may have on this process. We investigated the effect of early introduction of solid foods (before 6 months) whereas Bergtrom *et al*, looked at infants between 9 and 36 months of age [67]. It has been recently described that cessation of breast-feeding, rather than introduction of solid food, drives further maturation into adult like microbiota [15], and that breast milk may provide the gut microbiome with a greater plasticity that eases the transition into solid foods [69].

### Influence of antibiotic use on the infant gut microbiota during the first 6 months of life

Antibiotic use can cause an ecological disruption that might be even more pronounced when administered in early life, a period of critical development. Indeed, antibiotic exposure in early life is associated with numerous diseases later in life [4]. Antibiotic use was very low throughout the study; only 6 infants received antibiotics, which all occurred after 3 months. Infants receiving antibiotics had slightly lower total bacterial count (as measured by DAPI) (9.97 log10 cells/g of feces vs 10.44 log10 cells/g of feces, six months samples, p<0.001), lower *Bifidobacterium* (8.5 log10 cells/g of feces vs 9.7 log10 cells/g of feces, six months samples, p = 0.003), and *Staphylococcus* (4.9 log10 cells/g of feces vs. 5.7 log10 cells/g of feces, six months samples, p = 0.043). In addition, the probability to detect *B*. *longum* subsp. *longum* at 6 months was reduced by 7.4 times in case of antibiotic treatment (six months samples, p = 0.007).

The reduced colonization by bifidobacteria and staphylococci after antibiotic treatment is in line with previous findings [70–72]. We could not confirm the increase of *Enterococcus* or *Enterobacteria* following antibiotic treatment previously reported by others [54, 72], most probably due to the smaller sample size in our study.

### Other factors

Besides mode of delivery, mode of feeding, and antibiotic use, other factors associated with early exposure to microbes, such as farm exposure, place of birth, presence of siblings or pets are suggested to influence the early colonization process. For example, it has been previously described that infants born at hospital are more often colonized by *C*. *difficile* [13, 73]. In this study only 4.6% infants were born at home, and even though our results suggest that place of birth influences the colonization pattern of *B*. *breve* (higher probability to be detected when born at home; longitudinal model, p = 0.024), this should be further confirmed in larger studies.

Early exposure to microbes is suggested to decrease the risk of allergies later in life; and these associations are commonly attributed as “hygiene hypothesis” [74–76]. Although the mechanism of such a protective effect remains to be unraveled; it seems that contact with bacteria in early life plays a pivotal role in triggering the immune response and tolerance induction [14, 77]. Up to now, little evidence is available showing how the contact with a less sterile environment in early life, such as living in a rural environment, keeping indoor pets or having older siblings, differentially impact the gut microbiota composition.

#### A) Presence of older siblings or household pets

Exposure to pets has been associated with an over-representation of *Clostridium* species, *Veillonella*, *Peptostreptococcaceace* and *Coprococcus* and an under-representation of *Bifidobacterium*, or specifically *B*. *breve*, in the infant gut [78]. However, in our study a negative association was found only between exposure to pets and the colonization by the *L*. *reuteri* subgroup. The probability to detect *L*. *reuteri* subgroup was significantly higher in those infants not exposed to households pets (N = 90) (longitudinal model, p = 0.006) and those that were firstborns (N = 56) (longitudinal model, p<0.001). The latest was still significant at 6 months (six months samples, p = 0.013) (35.85% present in firstborns vs 6.15% if there were older siblings in the family as represented by the original data in S5 Fig). Our result suggests that colonization by bacteria belonging to *L*. *reuteri* subgroup is favored by a more sterile environment and exposure to formula feeding as discussed before, in which the exposure to microbes is limited. However, the relevance of this finding deserves further confirmation since the association between colonization by *L*. *reuteri* and increased allergy risk has not been established before, and in fact supplementation by a specific *L*. *reuteri* strain seems to increase the immune-regulatory capacity during infancy [79].

Besides the results on the *L*. *reuteri* subgroup described above, more correlations were found between prevalence of specific bacteria and the presence/absence of older siblings. Firstborn infants were 2.5 times more often colonized by *B*. *dentium* than those having older siblings (longitudinal model, p = 0.035), which was still significant at 6 months (six months samples, p = 0.003). On the contrary, infants with older siblings were 9 times more often colonized with *B*. *catenulatum* group (longitudinal model, p = 0.015) during the first 3 months of life, and the difference was still significant at 6 months (six months samples, p = 0.004) (Original data represented in S6 Fig).

**Fig 9:**
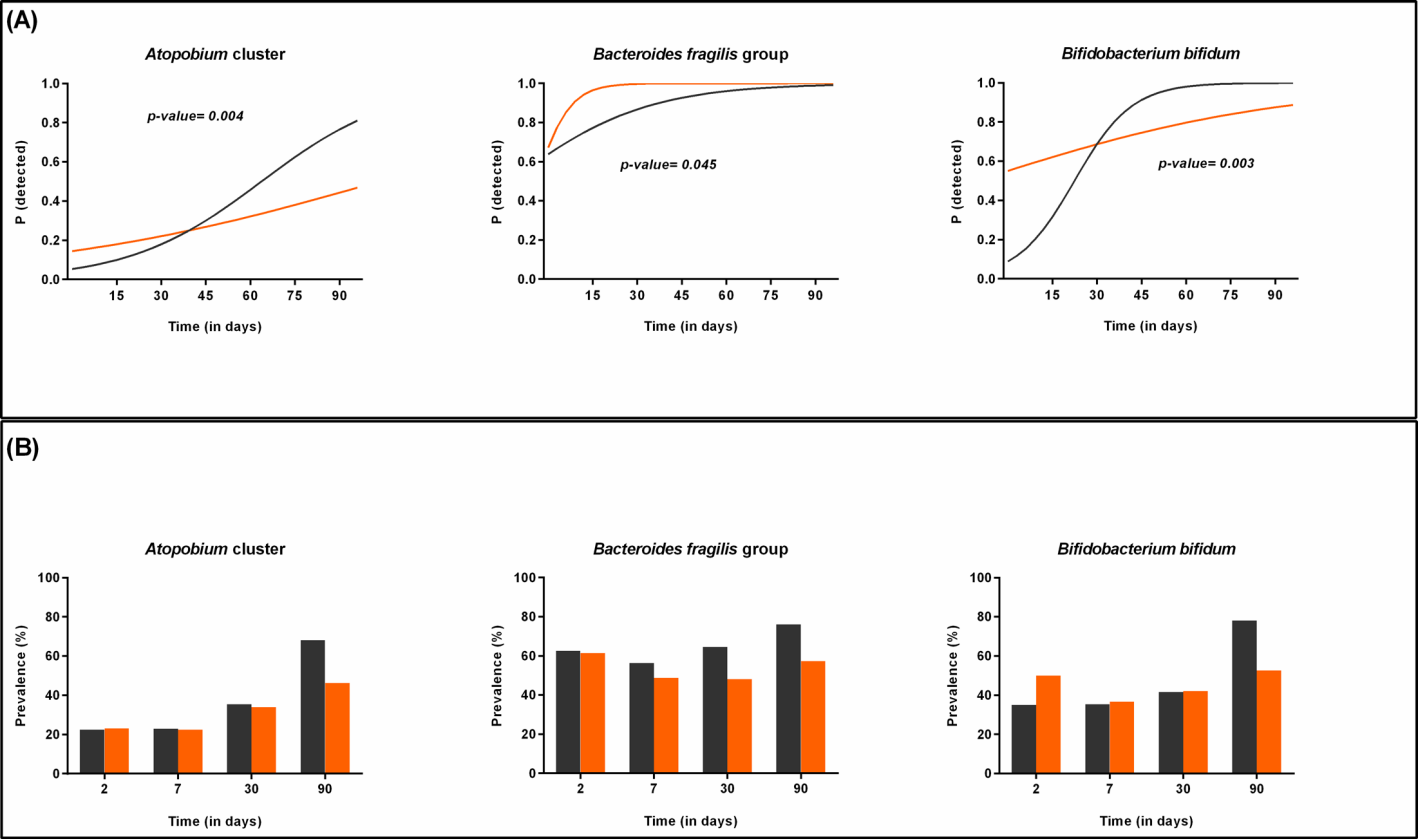
Bacterial groups or species influenced by the presence/absence of older siblings (I). (A) Probability to detect *Atopobium* cluster, *B*. *fragilis* group and *B*. *bifidum* in firstborn infants (orange) compared to those having older siblings (grey) during the first 3 months of life as calculated by the statistical model. (B) Prevalence of *Atopobium* cluster, *B*. *fragilis* group and *B*. *bifidum* in firstborn infants (orange) compared to those having older siblings (grey) during the first 3 months of life as represented by the original data.

Infants having older siblings were more often colonized by *B*. *fragilis* group (longitudinal model, p = 0.045) during the first 3 months of life and by *B*. *fragilis* subgroup at 6 months (six months samples, p = 0.041). In addition, the probability to detect *Atopobium* and *B*. *bifidum* in those infants, increased faster throughout the first three months of life when compared to firstborns (longitudinal model, p = 0.004 and p = 0.003, respectively) (Fig 9A). This difference was still significant for *B*. *bifidum* at 6 months of age (six months samples, p = 0.005) (84% vs 67.31% detected in firstborns as represented by the original data).

**Fig 10:**
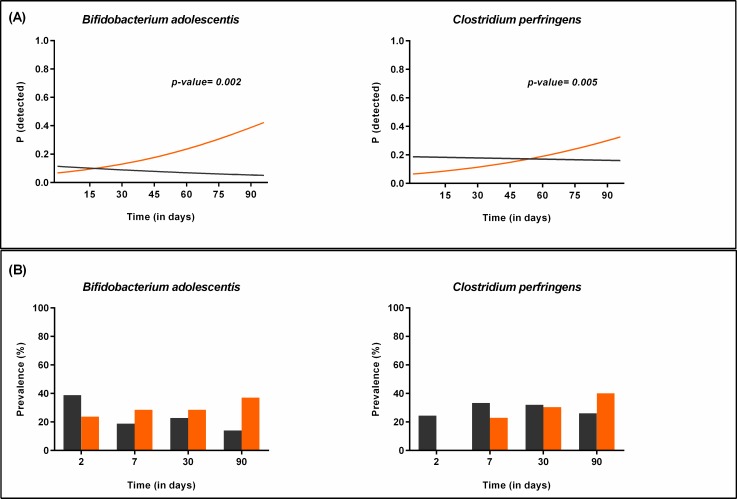
Bacterial groups or species influenced by the presence/absence of older siblings (II). (A) Probability to detect *B*. *adolescentis* and *C*. *perfringens* in firstborn infants (orange) compared to those having older siblings (grey) during the first 3 months of life as calculated by the statistical model. (B) Prevalence of *B*. *adolescentis* and *C*. *perfringens* in firstborn infants (orange) compared to those having older siblings (grey) during the first 3 months of life as represented by the original data.

On the other hand, the probability to detect *B*. *adolescentis* group and *C*. *perfringe*ns increased faster in firstborn infants during the first three months of life, when compared to those infants having older siblings (longitudinal model, p = 0.002 and p = 0.005, respectively; Fig 10A).

Several studies have reported that sibling number is associated with microbiota composition; first born infants are thought to be most often colonized by enterobacteria and clostridia, whereas the colonization by bifidobacteria, *Bacteroides* and *Lactobacillus* increase with an increasing number of siblings [13, 57, 80]. A positive correlation between numbers of older siblings and richness and bacterial diversity within the phyla of *Firmicutes* and *Bacteroidetes* at 18 months of age has been described recently [81].

Although we have not confirmed all previous findings, our results show that sibling number influences the microbiota composition at 6 months of age and describes differences at the species level. Whereas a reduced colonization by bifidobacteria has been reported before in first born infants [13, 57], we have shown that this is true for *B*. *bifidum* and *B*. *catenulatum* group, but not for *B*. *dentium* or *B*. *adolescentis* group, which were more often detected in first-born infants. Similarly, the previously reported reduced colonization by *Lactobacillus* in firstborn infants, seems not to be true for *L*. *reuteri* subgroup, which was most often detected in firstborn infants than in infants with older siblings even at 6 months of age. To our knowledge, this is the first time that *Atopobium* cluster has been shown to be dependent on number of siblings.

Our results provide new supporting evidence to the hygiene hypothesis, since the exposure to a less sterile environment (number of siblings) seems to favor the colonization by the type of bacteria that are reported to be absent in allergic infants in early life (*B*. *fragilis*, *B*. *bifidum* and *B*. *adolescentis)*, suggesting their role in the development of the immune system [82–85].

#### C. Gender

We included 56 boys (51.9%) and 52 girls (48.1%) in this study. At birth, boys had higher total bacterial count (as measured by DAPI) than girls (first defecation samples, p = 0.029). Girls were six times more frequently colonized by *L*. *ruminis* subgroup (first defecation samples, p = 0.003) at birth, 4 times by *L*. *gasseri* subgroup (longitudinal model, p<0.001) and 3 times by *L*. *reuteri* subgroup (longitudinal model, p<0.001) from 2 days to 3 months after birth.

To our knowledge, little is known about the influence of gender on the microbiota composition in early life. So far, only one study has reported gender differences in adults, showing a larger colonization by *Bacteroides* in males than in females [86]. Although puberty is thought to influence the microbiota composition due to hormonal changes, data supporting this idea is lacking in humans. Recently, it was shown through animal models that the gut microbiota of mice differs in males and females after puberty and that this trend is reversed by male castration [87–89]. Results from these animal models might point toward the difference in microbiota composition between male and female, as an explanation for a predisposition to certain immune diseases that are known to be gender-dependent (ie., diabetes type 1 in females or autism in males) [90].

Interestingly, we have found that *Lactobacillus*, a dominant genus in the vaginal microbiota and known to be regulated by female estrogens [91, 92], colonizes the gut of girls more often than boys in early life. Further research is needed to confirm this finding and elucidate whether microbiota and hormones can work together to influence gender bias in different diseases risk, and more importantly, whether changes in the gut microbiota in early life could modulate this susceptibility.

## Conclusions

To our knowledge this is one of the few studies using a statistical model to elucidate the complex process of early life colonization connecting longitudinal data and the different factors influencing this dynamic process. However, whereas currently 16S sequencing is often used to study the composition and diversity of complex microbial ecosystems, we chose to use a qPCR targeted approach, which allowed us to follow quantitatively the colonization of key taxa at species level.

This study contributes to understand not only the composition of the microbiota in early life, but also the succession process and the evolution of the microbial community as a function of time and important events or ecological drivers occurring the first 6 months of life.

Our results confirm that mode of delivery and type of feeding are important factors influencing this process, providing more detailed information about the timing of this influence and a deeper level of bacterial identification beyond genus. Additionally, this study reveals that other factors, which may have not yet been sufficiently studied or overlooked in the past; including presence of siblings, pets, and gender also influence this process. Even though the difference may be viewed as subtle, we need to further understand the implications of the differential effects observed and the association with potential health outcomes in the future. As expected, known early colonizers such as *Bifidobacterium* and/or specific *Bifidobacterium* species, are shown to be greatly influenced by most factors investigated in our study. Surprisingly, some of the known infant type bifidobacterial species, such *B*. *breve* and *B*. *longum* subsp. *infantis* were confirmed in this study to be early colonizers but were hardly dependent on the covariates studied. On the contrary, different *Lactobacillus* species are shown to be influenced by several covariates even though they are not normally dominant in the infant gut. In addition, our results also point out that *Bacteroides* is a key bacterial genus in the early colonization process, which is influenced by most factors included in the model to a larger extend than *Bifidobacterium*, which may indicate the need to consider strains belonging to this genus as potential probiotic interventions in infant nutrition in the future.

Our results also suggest that there is at least a certain degree of bacterial exposure *in utero* since 77.08% of the meconium samples contained bacterial DNA.

One needs to keep in mind that these results are generated using data driven covariate selection and therefore further studies are warranted to confirm these findings.

In summary, this study provides new insights that need to be taken into consideration when selecting the most relevant nutritional supplementation strategies to establish the microbiota composition in early life and potentially influence health later in life. According to our results these strategies may need to differ depending on the timing of supplementation, the environment in which the infant lives and maybe even whether it is a boy or a girl, which seems to point out to the need to develop personalized infant nutrition in the future.

## Supporting Information

S1 Fig Effect of type of feeding on the colonization by different bacteria. Prevalence of different bacterial groups significantly dependent on the type of feeding; at 2,7, 30 and 90 days of life. Graph representing original data in exclusive breastfed (green) or mix-fed infants (black).
(TIF)

S2 Fig Long-term effect of exclusive breastfeeding for 3 months (I). (A) Prevalence of L. *casei* subgroup and *B*. *adolescentis* at 6 months in infants that were exclusive breastfed for the first 3 months of life (green) or exposed to mix-feeding (black) as represented by the original data. (B) Prevalence of different organic acids at 6 months in infants that were exclusive breastfed for the first 3 months of life (black) or exposed to mix-feeding (grey) as represented by the original data.
(TIF)

S3 Fig Long-term effect of exclusive breastfeeding for 3 months (II). (A) Prevalence of *L*. *gasseri* subgroup from 3 to 6 months in infants that were exclusive breastfed for the first 3 months of life (green) or exposed to mix-feeding (black) as calculated by the statistical model. (B) Prevalence of *L*. *gasseri* subgroup at 3 and 6 months in infants that were exclusive breastfed for the first 3 months of life (green) or exposed to mix-feeding (black) as represented by the original data.
(TIF)

S4 Fig Effect of the introduction of solids foods. Effect of the introduction of solid foods on the prevalence of *C*.*coccoides* group and *Atopobium* cluster in the fecal sample taken at 6 months of age.
(TIF)

S5 Fig Prevalence of *L*. *reuteri* subgroup depending on the environment. (A) Prevalence of *L*. *reuteri* subgroup in infants not exposed to households’ pets (orange) or having pets at home (grey) over time. (B).Prevalence of *L*. *reuteri* subgroup in first-born infants (orange) and those having older siblings (grey) over time.
(TIF)

S6 Fig Bacterial groups or species influenced by the presence/absence of older siblings. Prevalence of *L*. *reuteri* subgroup, *B*. *dentium* and *B*. *catenulatum* group in firstborn infants (orange) and those having siblings (grey) over time,
(TIF)

S1 Table Mean counts and prevalence of intestinal bacteria in feces of infants at birth, 2, 7, 30, 90 and 180 days. (PDF)
