# Supplementary figures and images for: Early-Life Events, Including Mode of Delivery and Type of Feeding, Siblings and Gender, Shape the Developing Gut Microbiota
Source: PLoS One. 2016 Jun 30;11(6):e0158498. doi: 10.1371/journal.pone.0158498 (PMC4928817; doi:10.1371/journal.pone.0158498)

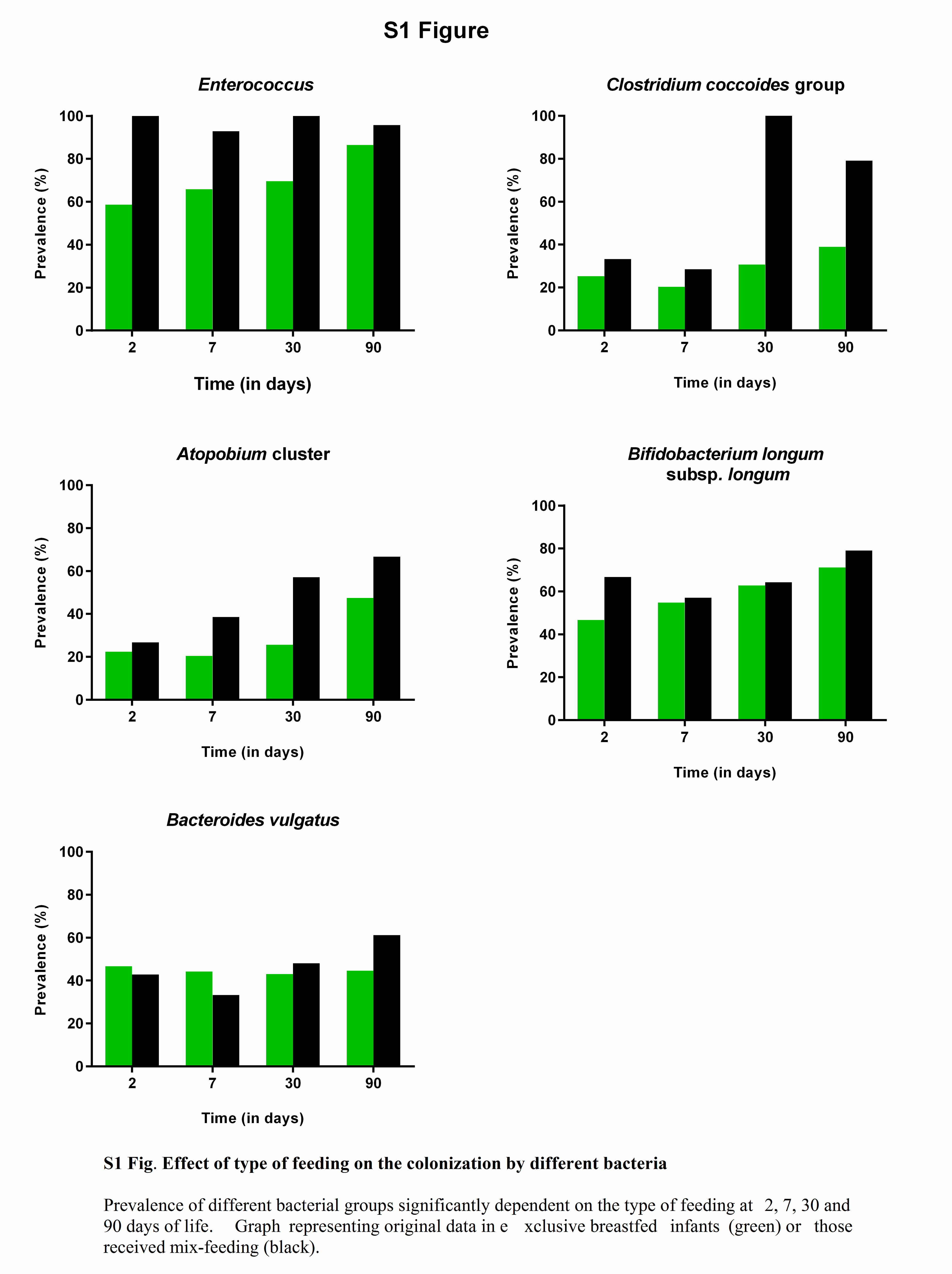

Supplement: S1 Fig — Prevalence of different bacterial groups significantly dependent on the type of feeding; at 2,7, 30 and 90 days of life. Graph representing original data in exclusive breastfed (green) or mix-fed infants (black). (TIF) [file pone.0158498.s001.tif]

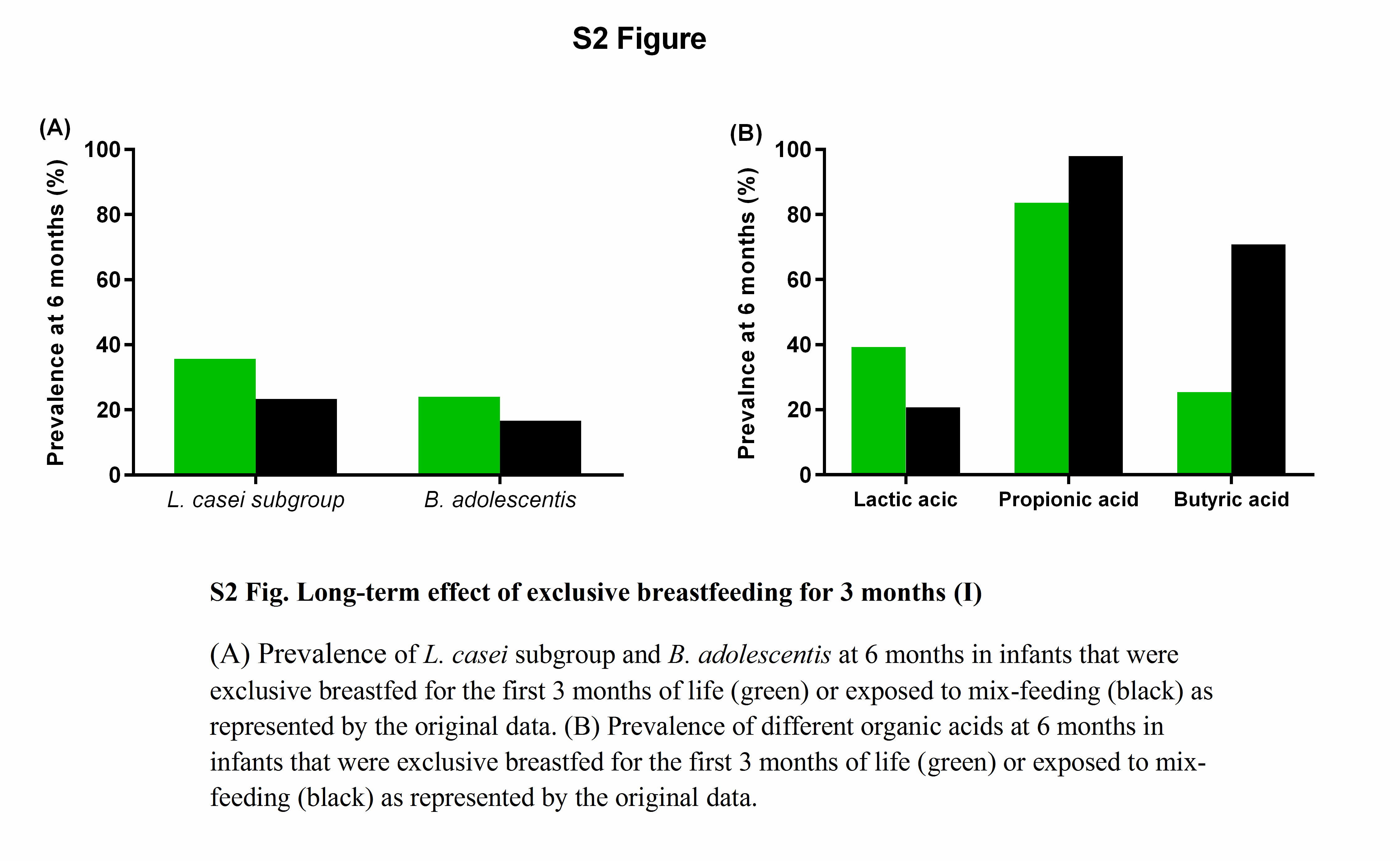

Supplement: S2 Fig — (A) Prevalence of L. casei subgroup and B. adolescentis at 6 months in infants that were exclusive breastfed for the first 3 months of life (green) or exposed to mix-feeding (black) as represented by the original data. (B) Prevalence of different organic acids at 6 months in infants that were exclusive breastfed for the first 3 months of life (black) or exposed to mix-feeding (grey) as represented by the original data. (TIF) [file pone.0158498.s002.tif]

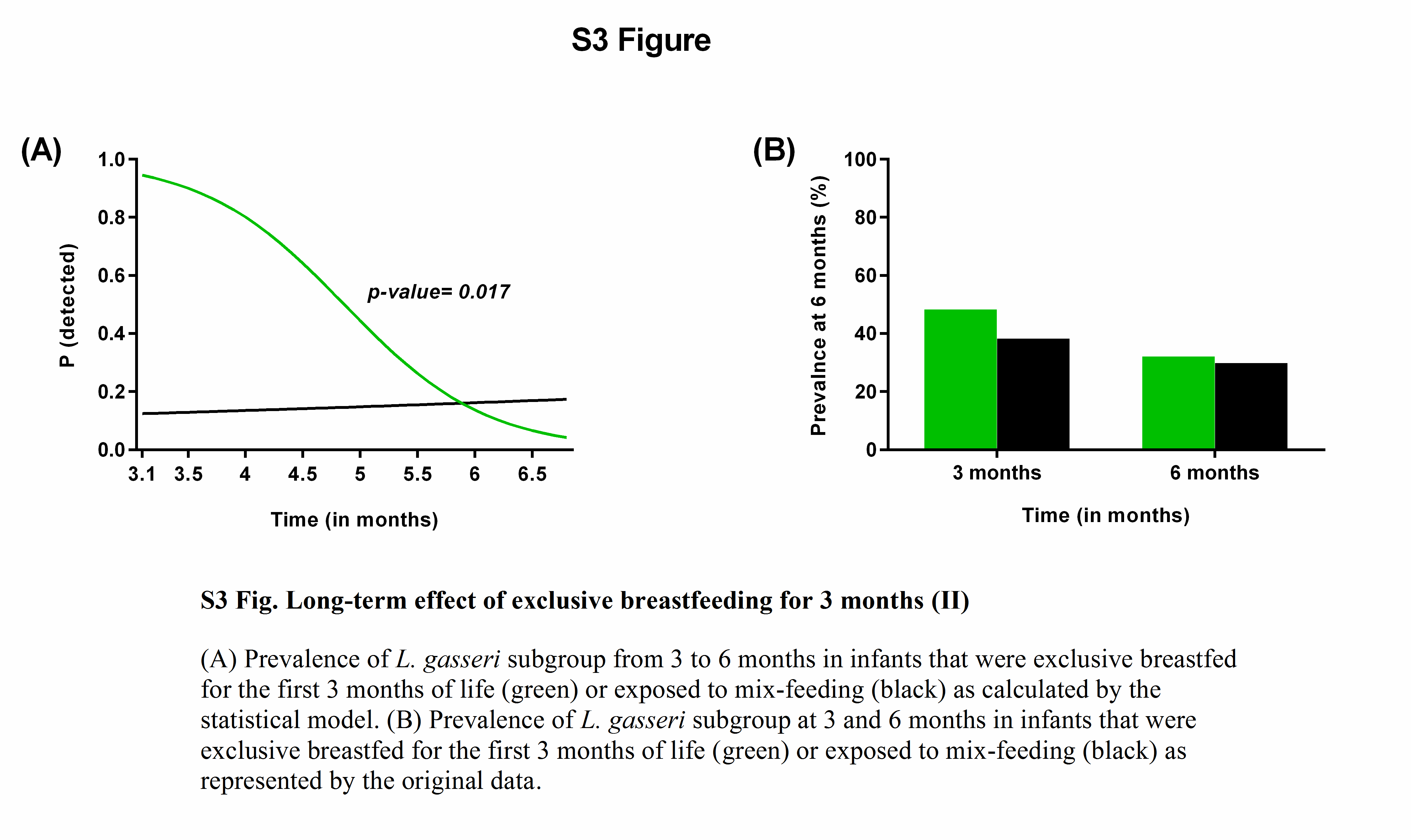

Supplement: S3 Fig — (A) Prevalence of L. gasseri subgroup from 3 to 6 months in infants that were exclusive breastfed for the first 3 months of life (green) or exposed to mix-feeding (black) as calculated by the statistical model. (B) Prevalence of L. gasseri subgroup at 3 and 6 months in infants that were exclusive breastfed for the first 3 months of life (green) or exposed to mix-feeding (black) as represented by the original data. (TIF) [file pone.0158498.s003.tif]

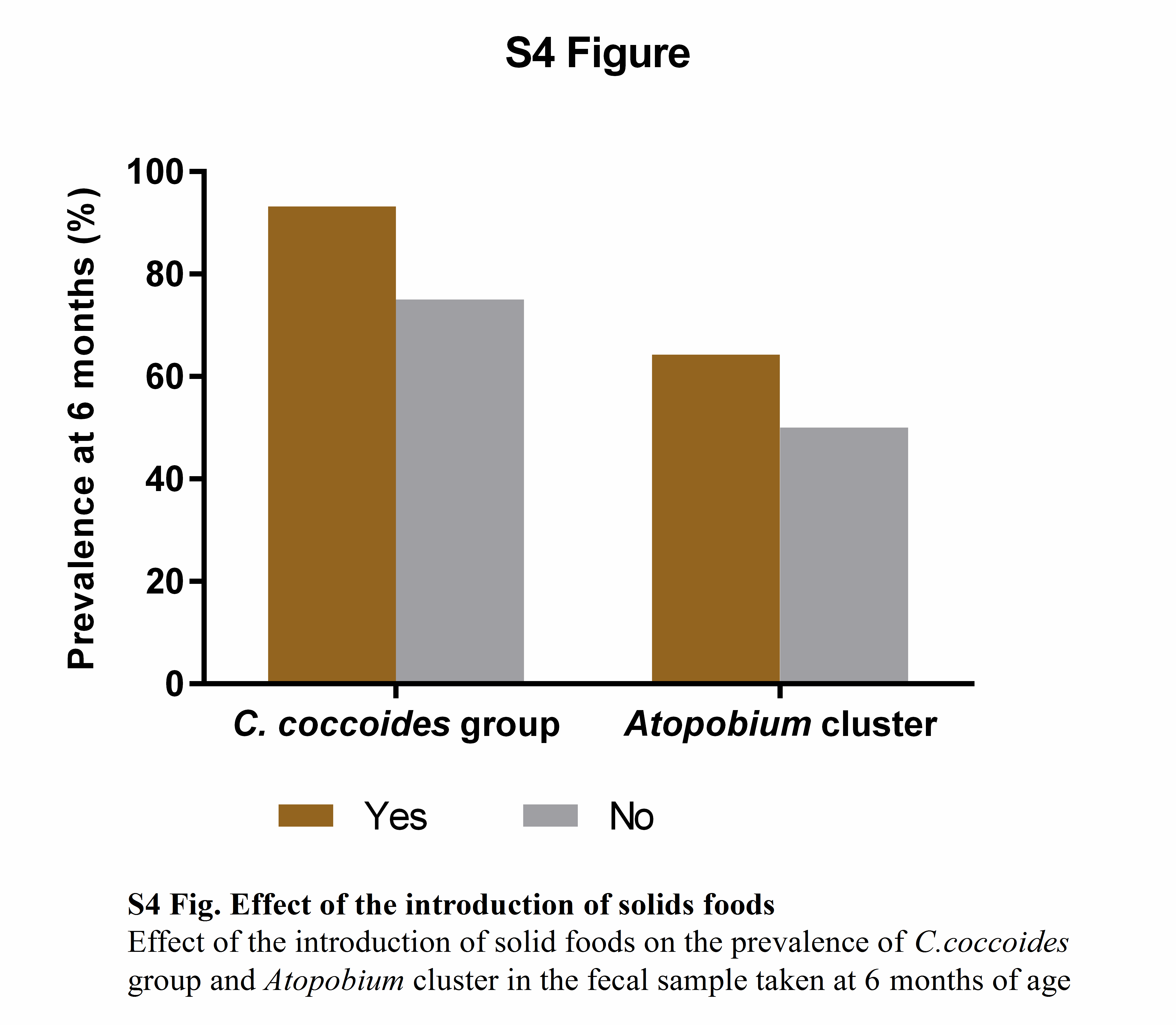

Supplement: S4 Fig — Effect of the introduction of solid foods on the prevalence of C.coccoides group and Atopobium cluster in the fecal sample taken at 6 months of age. (TIF) [file pone.0158498.s004.tif]

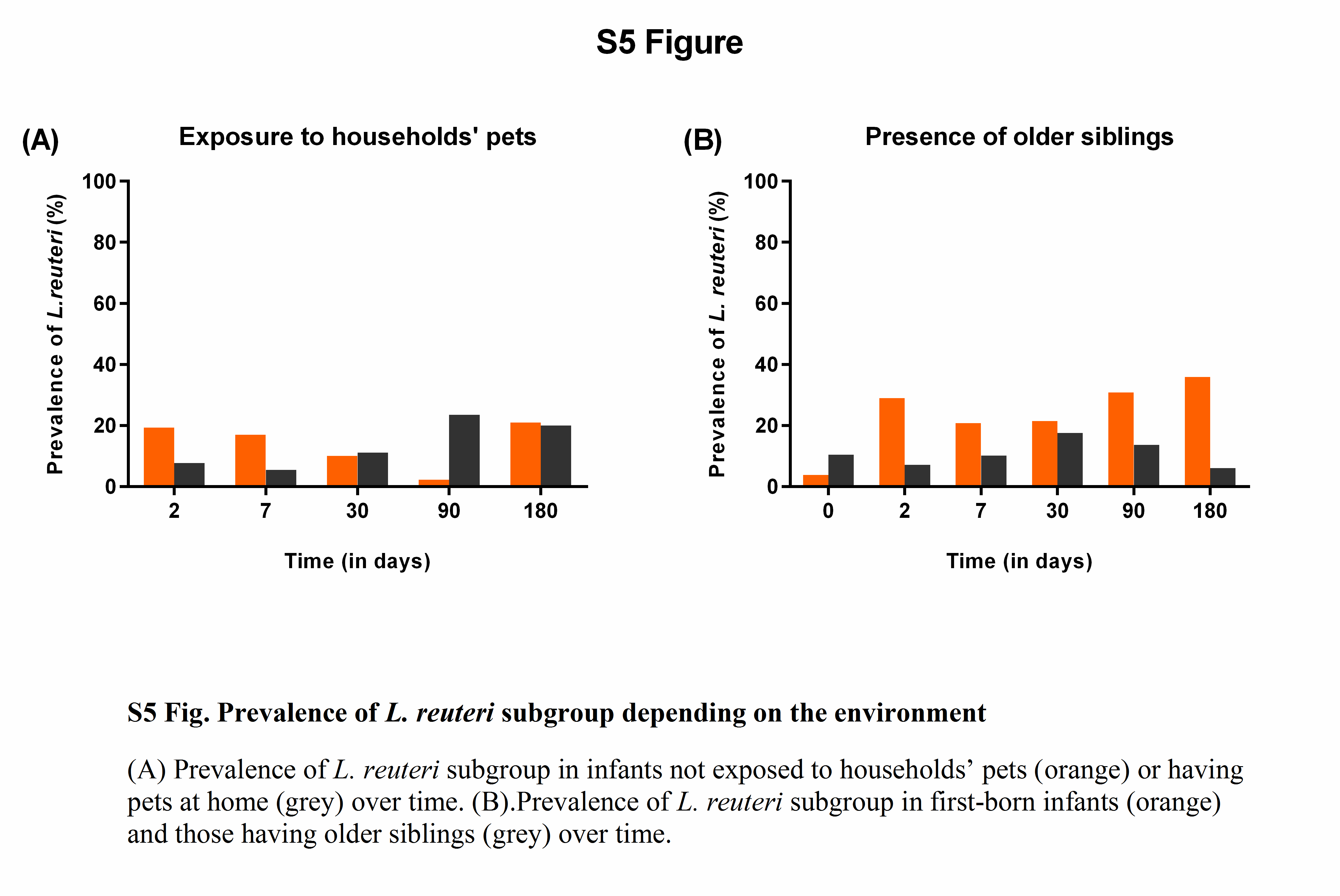

Supplement: S5 Fig — (A) Prevalence of L. reuteri subgroup in infants not exposed to households’ pets (orange) or having pets at home (grey) over time. (B).Prevalence of L. reuteri subgroup in first-born infants (orange) and those having older siblings (grey) over time. (TIF) [file pone.0158498.s005.tif]

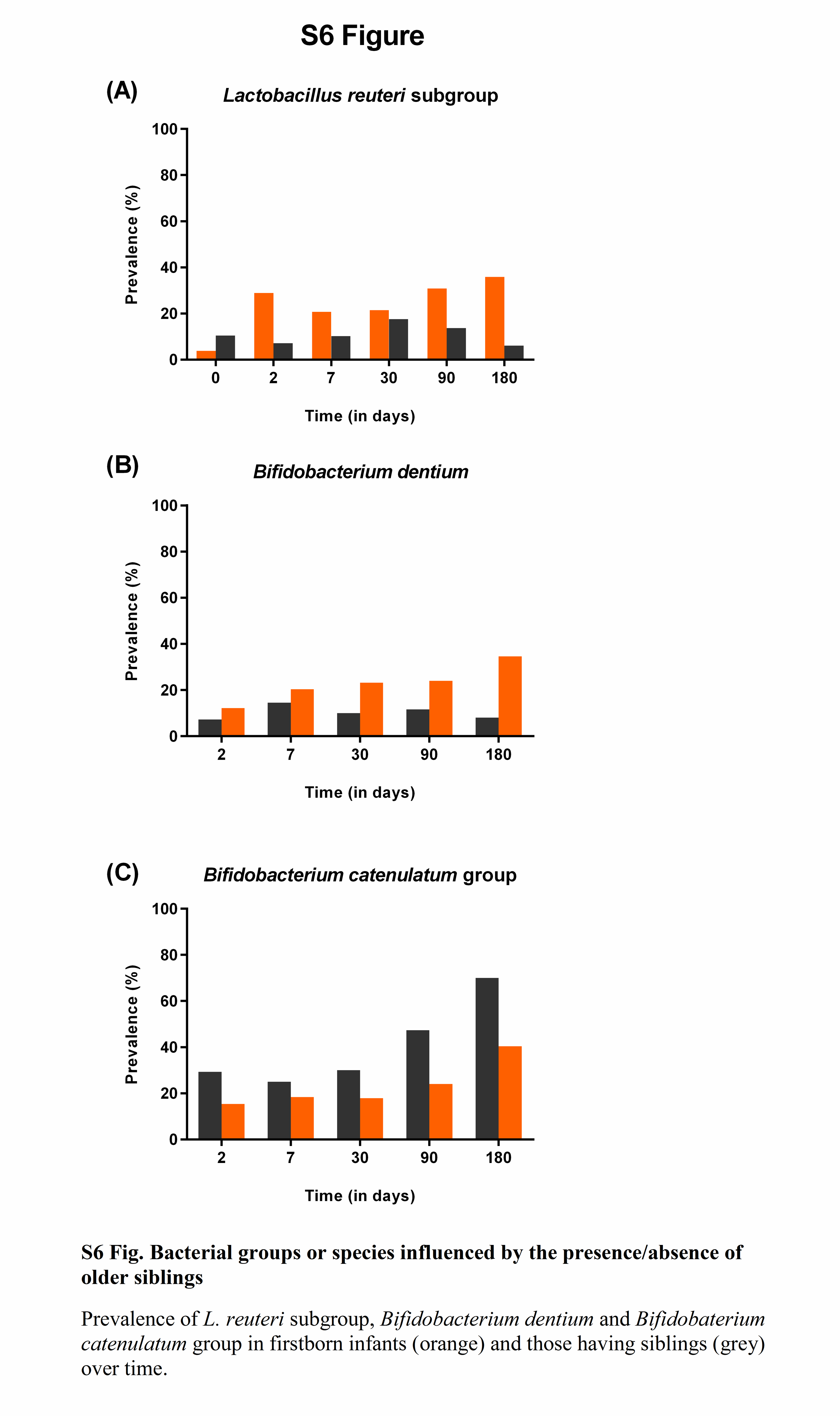

Supplement: S6 Fig — Prevalence of L. reuteri subgroup, B. dentium and B. catenulatum group in firstborn infants (orange) and those having siblings (grey) over time, (TIF) [file pone.0158498.s006.tif]
