# Supplementary material for: Early-Life Events, Including Mode of Delivery and Type of Feeding, Siblings and Gender, Shape the Developing Gut Microbiota
Source: PLoS One. 2016 Jun 30;11(6):e0158498. doi: 10.1371/journal.pone.0158498 (PMC4928817; doi:10.1371/journal.pone.0158498)
Supplement: S1 Table — (PDF) [file pone.0158498.s007.pdf]

S1 Table . Mean counts and prevalence of intestinal bacteria in feces of infants at birth, 2, 7, 30, 90 and 180 days

	**Meconium (96)**		**2 days (80)**		**7 days (98)**		**30 days (107)**		**90 days (105)**		**180 days (103)**	
	Counts*	(%)	Counts*	(%)	Counts*	(%)	Counts*	(%)	Counts*	(%)	Counts*	(%)
Clostridium coccoides	6.71±0.36	3.13	8.19±1.04	26.25	7.63±1.16	22.45	8.22±1.31	35.85	8.69±0.75	58.10	8.94±1.00	89.22
Clostridium leptum	7.50±0.59	3.13	7.56±0.52	22.50	7.71±0.59	11.22	7.76±0.49	15.09	7.95±0.69	34.29	8.53±0.89	66.67
Bacteroides fragilis subgroup	7.41±1.00	10.42	8.49±0.87	62.50	8.31±1.11	52.04	8.52±0.92	55.66	8.72±0.77	65.71	9.04±0.73	76.47
Bifidobacteria	7.69±1.13	16.17	7.07±3.07	97.50	8.61±1.93	100.00	8.94±2.17	100.00	9.67±1.07	100.00	9.79±0.87	100.00
Atopobium	5.57	1.04	8.23±0.92	22.50	8.09±0.8	23.47	8.06±0.81	33.96	8.30±0.81	57.14	8.06±0.64	71.57
Prevotella	-	-	7.47±1.46	2.50	6.78±0.34	5.10	6.93±0.65	8.49	7.12±0.83	11.43	7.83±1.08	21.57
Lactococcus lactis	-	-	5.48	1.25	6.48	1.03	6.74±0.48	1.89	5.16±0.75	2.83	6.68±0.44	5.83
Clostridium perfringens	6.23±1.50	4.17	4.61±1.86	12.50	5.74±1.61	28.87	5.26±1.69	31.78	4.74±1.62	33.96	4.31±1.33	35.92
Enterobacteriaceae	6.95±1.33	37.50	8.21±1.44	66.25	8.37±1.23	79.38	8.42±0.9	93.46	8.26±0.73	98.11	7.69±0.95	97.09
Enterococcus	6.22±2.09	19.79	7.12±1.73	62.50	6.62±1.81	73.20	6.62±1.62	77.57	7.35±1.34	91.51	6.95±1.59	98.06
Staphylococcus	5.77±1.47	67.71	7.78±1.03	100.00	8.60±0.60	100.00	7.58±0.89	100.00	6.56±0.87	100.00	5.55±0.84	88.35
Lactobacillus ruminis	4.23±1.31	20.83	3.66±0.55	22.50	4.74±1.29	24.74	4.72±1.84	23.00	4.61±1.77	24.53	4.54±2.29	5.83
Lactobacillus casei	4.64±1.05	2.08	6.07±1.81	5.00	5.83±1.68	11.34	5.92±1.56	25.23	6.59±1.73	38.68	6.34±1.70	30.10
Lactobacillus gasseri	4.82±0.75	15.63	5.18±0.9	43.75	5.76±1.5	38.14	6.84±1.47	45.79	6.11±1.23	42.45	5.24±1.21	31.07
Lactobacillus reuteri	3.69±0.80	7.29	4.07±0.95	17.50	4.22±1.24	15.46	5.41±1.65	19.27	5.37±1.51	22.64	4.82±1.4	21.36
Lactobacillus sakei	3.72±0.62	15.63	3.78±0.64	28.75	3.75±0.74	37.11	3.73±0.88	19.63	3.78±0.67	32.08	3.72±0.68	38.83
Lactobacillus plantarum	3.99±2.31	5.21	8.15	1.25	3.94±0.61	4.12	4.49±1.08	6.54	3.50±0.31	4.72	3.72±0.56	28.16
Lactobacillus fermentum	-	0.00	-	0.00	7.09±1.99	3.09	6.83±1.92	8.41	7.21±1.32	6.60	7.58±1.65	4.85
Bacteroides caccae	7.13	1.04	7.73±0.46	13.75	7.77±0.83	4.12	8.39±0.66	8.41	7.98±0.72	16.04	8.4±0.65	20.59
Bacteroides eggerthi	-		-		–	-	-	0.00	-	0.00	7.45	0.98
Bacteroides fragilis subsp.	8.08±0.54	2.08	7.95±0.86	16.25	8.74±0.63	9.28	8.3±0.99	10.28	8.35±0.79	19.81	8.31±0.64	32.35
Bacteroides ovatus	8.13	1.04	7.95±0.54	11.25	8.25±0.79	13.40	8.14±0.81	18.69	8.07±0.53	19.81	8.48±0.66	24.51
Bacteroides theta	7.08	1.04	7.65±0.5	7.50	8.05±0.74	4.12	7.85±0.89	11.21	8.17±0.55	10.38	7.92±0.59	17.65
Bacteroides uniformis	-		8.1±0.54	17.50	8.22±0.73	11.34	8.09±0.64	16.82	7.93±0.65	26.42	8.25±0.63	34.31
Bacteroides vulgatus	7.54±0.78	8.33	8.31±0.99	52.50	7.86±0.99	43.30	8.07±0.9	43.93	8.10±1.00	51.89	8.81±0.72	58.82
Bifidobacterium adolescentis	7.07±1.04	9.38	7.75±1.12	30.00	7.95±1.39	23.71	8.13±1.57	25.23	8.78±1.27	25.47	8.85±1.27	20.59
Bifidobacterium animalis	7.99±0.26	2.08	-	0.00	7.78±0.8	10.31	7.61±0.98	13.08	7.76±1.06	17.92	7.55±0.92	41.18
Bifidobacterium bifidum	7.59±1.55	11.46	8.73±1.41	42.50	9.47±0.79	36.08	9.36±0.96	42.06	9.25±0.81	64.15	8.90±0.72	75.49
Bifidobacterium breve	8.35±0.65	2.08	8.40±1.36	17.50	9.26±1.24	18.56	9.61±1.01	39.25	9.39±1.02	54.72	9.03±1.03	78.43
Bifidobacterium catenulatum	7.60±0.33	2.08	8.54±0.73	22.50	8.65±0.92	21.65	8.45±0.68	23.36	8.70±0.87	34.91	8.55±0.97	54.90

Bifidobacterium longum	7.99±1.14	8.33	8.28±0.81	48.75	8.78±0.85	55.67	8.57±1	62.62	8.59±1.08	75.47	8.54±0.85	83.33
Bifidobacterium infantis	7.08	1.04	7.52	1.25	8.41±0.92	5.15	8.08±1.14	7.48	8.89±0.97	9.43	8.31±1.09	20.59
Bifidobacterium dentium	-		7.03±0.71	10.00	7.62±1.41	18.56	7.81±1.17	16.82	7.24±0.78	17.92	7.09±0.80	21.57
Total Bacteria (DAPI)	8.32±1.11	32.29	9.82±0.59	100.00	10.07±0.40	100.00	10.29±0.38	100.00	10.54±1.42	100.00	10.38±0.45	100.00

*Data are expressed as the means (log10 cel and standard deviations

(X) Number of subjects
